# Performance of technical trading rules: evidence from Southeast Asian stock markets

**DOI:** 10.1186/s40064-015-1334-7

**Published:** 2015-09-25

**Authors:** Piyapas Tharavanij, Vasan Siraprapasiri, Kittichai Rajchamaha

**Affiliations:** College of Management (CMMU), Mahidol University, Bangkok, Thailand

**Keywords:** Technical analysis, Trading system, Trading rule, G12, G14

## Abstract

This paper examines the profitability of technical trading rules in the five Southeast Asian stock markets. The data cover a period of 14 years from January 2000 to December 2013. The instruments investigated are five Southeast Asian stock market indices: SET index (Thailand), FTSE Bursa Malaysia KLC index (Malaysia), FTSE Straits Times index (Singapore), JSX Composite index (Indonesia), and PSE composite index (the Philippines). Trading strategies investigated include Relative Strength Index, Stochastic oscillator, Moving Average Convergence-Divergence, Directional Movement Indicator and On Balance Volume. Performances are compared to a simple Buy-and-Hold. Statistical tests are also performed. Our empirical results show a strong performance of technical trading rules in an emerging stock market of Thailand but not in a more mature stock market of Singapore. The technical trading rules also generate statistical significant returns in the Malaysian, Indonesian and the Philippine markets. However, after taking transaction costs into account, most technical trading rules do not generate net returns. This fact suggests different levels of market efficiency among Southeast Asian stock markets. This paper finds three new insights. Firstly, technical indicators does not help much in terms of market timing. Basically, traders cannot expect to buy at a relative low price and sell at a relative high price by just using technical trading rules. Secondly, technical trading rules can be beneficial to individual investors as they help them to counter the behavioral bias called disposition effects which is the tendency to sell winning stocks too soon and holding on to losing stocks too long. Thirdly, even profitable strategies could not reliably predict subsequent market directions. They make money from having a higher average profit from profitable trades than an average loss from unprofitable ones.

## Background

Technical analysis involves making investment decisions based on past trading data. It aims to establish buying and selling rules that maximize profits and still control risks of loss. Unfortunately, according to the efficient market hypothesis (EMH), this endeavor is ultimately futile. The EMH states that all available and relevant information are already incorporated in security prices. As technical analysis uses only current and past trading data, it is not possible to obtain abnormal positive returns by applying these technical trading rules. If investors could make money from applying these trading rules, this would indicate that the market is inefficient. Therefore, the question of whether technical trading rules can consistently generate profits becomes an empirical issue concerning efficiency of actual markets.

The case of Southeast Asian stock market is interesting since both Bessembinder and Chan (1998) and Ratner and Leal (1999) find that trading rules are successful in predicting stock price movement in Southeast Asian markets. More recently, Yu et al. (2013) also find that technical trading rules have predictive power particularly in emerging markets of Malaysia, Thailand, Indonesia, and the Philippines but to a much lower extent in a more mature market of Singapore. However, they find that transaction costs can eliminate trading profits in most markets, except Thailand.

This study revisits this important issue by expanding the scope of trading rules. Instead of focusing on moving average rules or trading range breakout rules like in previous research (e.g. Yu et al. 2013), this paper focuses on popular technical indicators reported in the media and applied in actual markets by technical analysts. In addition, we study the odds of profitable or unprofitable trades and the associated returns. We also investigate the performances of technical trading rules with optimized parameters compared to those from standard parameters. These issues are usually overlooked in previous studies.

Specifically, this paper examines and conducts formal statistical tests on the profitability of various technical trading rules when applied to five Southeast Asian stock markets. Profitability is defined as the ability to earn annualized returns in excess of a simple Buy-and-hold (BH) strategy. The data cover a period of 14 years from January 2000 to December 2013. Trading strategies studied include Relative Strength Index (RSI), Stochastic oscillator (STOCH), Moving Average Convergence-Divergence (MACD), Directional Movement Indicator (DMI) and On Balance Volume (OBV).

Our results suggests different levels of market efficiency among Southeast Asian stock markets. On one hand, technical trading strategies give statistically significant returns and positive net returns after transaction costs in an emerging stock market of Thailand. On the other hand, no technical trading strategies investigated generate statistically significant returns in a more mature stock market of Singapore. In other markets, the technical trading rules also generate statistical significant returns, however, after taking transaction costs into account, most do not generate positive net returns.

We also find that profitable technical trading strategies do not reliably predict subsequent market movements. Instead, they make money from letting the profits to run in profitable trades while minimizing loss in unprofitable ones.

With optimized parameters, we find that unprofitable strategies still remain unprofitable. In contrast, profitable strategies become much more profitable. We notice that there is no universal optimal parameters. The optimized parameters are market specific and can differ a lot from standard parameters. Interestingly, optimized parameters do not improve the probability of profitable trades as the percentage of profitable trades remain stable.

## Literature review

According to the efficient market hypothesis (EMH), which states that asset prices incorporate all available and relevant information, it is impossible to make risk-adjusted profits by trading on the past trading data. Therefore, any attempt to make profits by technical analysis is ultimately futile. However, even from a theoretical perspective, the EMH has been increasingly challenged.

Grossman and Stiglitz (1980) shows that if obtaining and processing information is costly, then the market price cannot incorporate all available relevant information because otherwise there would be no incentive to obtain and process costly information in the first place. They conclude that the market cannot be fully efficient. Later on, behavioral models are developed to explain how profitable trading opportunities based on past trading data can still exist. Basically, these types of models show that price adjusts slowly to new information due to noise trading, feedback trading or herding behavior.

Brown and Jennings (1989) develop a noisy rational expectations model, in which the current price does not fully reveal private information. Thus, historical prices can help predicting future prices. In a feedback model (DeLong et al. 1990), there are noise traders who irrationally trade on noise and follow a positive feedback strategy by buying when prices rise and selling when prices fall. As a result, an asset can be overpriced or underpriced even more by noise traders at least in the short run. Shleifer and Summers (1990) even suggest that technical trading based on noises can make profits even in the long run. In their herding model, Froot et al. (1992) demonstrate that herding behavior of short-horizon traders can lead to informational inefficiency. The reason is that short-horizon traders would make profits only when they process the same information, which is not necessarily relevant to asset values. Therefore, these short-term traders (herders) would follow the same technical indicators to make profits.

Park and Irwin (2007) provide a review of empirical studies on the issue of trading rule profitability. In their review, modern studies (papers published from 1988 to 2004) indicate that technical trading strategies consistently generate profits at least until the early 1990s. Among a total of 95 studies, 56 studies find profitability of technical trading, 20 studies obtain negative results and 19 studies indicate mixed results. The studies, which find profitability of trading rules, are Sullivan et al. (1999), Lo et al. (2000), and Kavajecz and Odders-White (2004). However, Brock et al. (1992), Bessembinder and Chan (1998), Ready (2002), Marshall et al. (2008) show that transaction costs would eliminate any trading profits. More recently, Bajgrowiczy and Scaillet (2012) point out that the profitability of technical analysis has declined over time.

In the case of emerging markets, there are more studies that find profitability of technical trading rules. Ratner and Leal (1999) examines the potential profit of technical trading rules in ten emerging equity markets in Latin America and Asia from 1982 to 1995. They find that Taiwan, Thailand and Mexico emerge as markets where technical trading strategies may be profitable.

Interestingly, papers that study emerging markets in Asian markets tend to find profitability of technical trading rules. For instance, Lento (2006), which studied performance of nine technical trading rules in eight Asian-Pacific equity markets from 1987 to 2005, find that technical trading rules seem to be profitable in six Asian markets. In another study, Ming–Ming and Siok–Hwa (2006) examine the profitability of trading rules in nine Asian stock market indices from 1988 to 2003. Their results give strong support for trading rules in the China, Thailand, Taiwan, Malaysian, Singaporean, Hong Kong, Korean, and Indonesian stock markets.

More recently, Yu et al. (2013) study whether simple trading rules like moving average and trading range breakout rules can outperform a simple buy-and-hold strategy. Their samples are Southeast Asian stock markets from 1991 to 2008. They find profitability of trading rules in the stock markets of Malaysia, Thailand, Indonesia, and the Philippines, but not in the stock market of Singapore. However, they also observe that except in Thailand, trading rules cannot beat a buy-and-hold strategy after transaction costs.

## Trading rules

This study investigates five popular technical indicators. The first two, namely RSI and STOCH, are based on the contrarian idea. Basically, when the stock is overbought (oversold), the price tends to decrease (increase) afterward. The next two, namely MACD and DMI, are trend-following indicators. By riding a trend, technical analysis asserts that investors could make profits. The last indicator, OBV, is a volume-based indicator. It shows whether volume is flowing into or out of a security.

Each indicator is characterized by a number of parameters called “Ns”, i.e. N1, N2, N3 and so forth. The “standard” values for these parameters are the most popular numbers used by technical traders as reported in Colby (2003). On the other hand, the “optimal” values for these parameters are the ones that maximize net profits.

The simulated portfolio set up for testing each trading rule follow the following rules. When there are no signals, the entire portfolio consists of only cash deposit with no interest just to be conservative. For long-only strategies, if there is a buy signal on any particular day, then our simulated investor would use the entire cash to buy stocks the following trading day at the opening price. He will hold these stocks as long as there is no sell signal. When he gets a sell signal on any particular day, he will liquidate all stocks holding into cash on the following trading day at the opening price. For short-only strategies, the rules are similar but with opposite transactions. All long and short positions are closed at the end of the simulation. Transaction costs are ignored at this stage as their impact would be investigated with the round-trip breakeven costs later.

The detail of each trading rule is as follow.

### Relative Strength Index (RSI)

The RSI measures the current and historical strength or weakness of stock or market price movements based on closing prices of a recent trading period. Stocks which have had stronger positive changes have a higher RSI than stocks which have had stronger negative changes.

The idea behind is that when price moves up very rapidly, at some point it is considered overbought. Likewise, when price falls very rapidly, at some point it is considered oversold. In either case, a reversal is to be expected.

The RSI ranges from 0 to 100, with high and low levels marked at 70 and 30, respectively. Traditionally, RSI readings greater than the 70 level are considered to be in an overbought territory (Bearish signal), whereas RSI readings lower than the 30 level are considered to be in an oversold territory (Bullish signal). In between the 30 and 70 level is considered neutral, with the 50 level a sign of no trend.

Mathematically, the RSI is calculated by the following steps. First, calculate the “U” and “D” variables. The variable “U” equals an increase in price when a price moves up and zero otherwise. In opposite, the variable “D” equals an (absolute) decrease in price when a price moves down and zero otherwise. Second, compute average “U” (Ua) and average “D” (Da) by doing exponential moving averages of “U” and “D” over “N1” periods, respectively. The RSI is defined by the following equation.

$$RSI(P,N1) = \frac{Ua(P,N1)}{[Ua(P,N1) + Da(P,N1)]} \times 100$$

*P*_*t*_ is the closing price at time “t”

The standard value for “N1” is 14 (Colby 2003). This paper also searches for an optimal parameter value and then compares results with that from a standard parameter.

The buy signal to enter a long position (or to cover a prior short position) is generated when the RSI is in an oversold territory (RSI < 30). On the other hand, the sell signal to enter a short position (or to close a prior long position) is generated when the RSI is in an overbought territory (RSI > 70).

### Stochastic oscillator (STOCH)

The stochastic oscillator is an indicator that uses support and resistance levels in an attempt to anticipate price turning points. Its value is determined by the location of a current price in relation to its price range over a period of time.

Basically, the current security’s price is expressed as a percentage of this range with 0 % indicating the bottom of the range and 100 % indicating the upper limits of the range over the time period covered. The idea behind is that prices tend to close near the extremes of the recent range before turning points. Traditionally, Stochastic Oscillator readings greater than the 80 level are considered to be in an overbought territory (Bearish signal), whereas readings lower than the 20 level are considered to be in an oversold territory (Bullish signal).

Mathematically, the stochastic oscillator (%K) is calculated by the following formula.

$$\% K(N1,N2) = \frac{{\sum\limits_{i = 0}^{N2} {[P_{t - i} - LL_{t - i} \, ({\text{N}}1)]} }}{{\sum\limits_{i = 0}^{N2} {[HH_{t - i} \, ({\text{N}}1) - LL_{t - i} \, ({\text{N}}1)]} }} \times 100$$

*P*_*t*_ is the closing price at time “t”, *LL*(*N*1) is the lowest low price of previous N1-period, *HH*(*N1*) is the highest high price of previous N1-period and *N2* is the averaging period of %K.

The standard values for “Ns” are 5 days (N1) and 1 day (N2) (Colby 2003). This paper also searches for optimal parameter values and then compares results with that from standard parameters.

The buy signal to enter a long position (or to cover a prior short position) is generated when the stochastic oscillator is in an oversold territory (%K < 20). On the other hand, the sell signal to enter a short position (or to close a prior long position) is generated when it is in an overbought territory (%K > 80).

This paper also tests another variant of a trading rule based on STOCH. Basically, instead of using a fixed band, the buy signal is generated when %K line crosses above %D line (moving averages of %K), while the sell signal is generated when %K line crosses below %D line. Let us call this trading rule “stochastic oscillator crossing its own moving average” (STOCH-D).

Mathematically, the moving average (%D) of stochastic oscillator (%K) is calculated by the following formula.

$$\% D = EMA\;[\% K(N1,N2),N3]$$

*N3* is the averaging period of %D. *EMA* stands for exponential moving average.

The standard values for “Ns” are 5 days (N1), 1 day (N2) and 3 days (N3) (Colby 2003). Again, we also search for optimal parameter values and then compare results with that from standard parameters.

### Moving Average Convergence-Divergence (MACD)

The MACD is a difference between two exponential moving averages (EMA) of the closing price. A slower EMA is subtracted from a faster EMA. Then the MACD itself is smoothed again with an even faster EMA to get the MACD’s Signal Line. The difference between MACD and MACD’s Signal Line is a MACD’s Histogram.

To calculate MACD, first we must calculate EMA of close prices. Generally, we write EMA as a function of N Periods. For example, EMA (P,N) means the exponential moving averages of close prices (P) over N days.

Mathematically, the EMA is calculated by the following equation.

$$\begin{aligned} &EMA_{t} = EMA_{t - 1} + \alpha (P_{t} - EMA_{t - 1} ) = \alpha P_{t} + (1 - \alpha )EMA_{t - 1} \hfill \\ & \alpha = \frac{2}{(N + 1)} \hfill \\ \end{aligned}$$

*P*_*t*_ is the closing price at time “t”, *N* is the number of days and EMA stands for exponential moving average. *α* is the weight given to the most recent observation. Basically, it is a smoothing factor (the lower, the smoother EMA). 1 – α is the weight given to the latest smoothed variable.

We start the recursion by setting EMA_1_ = SMA(P,N), which is a simple average of close prices over N days.

The smoothing factor ($\alpha$) is chosen so as to give the same “average age” of the data as that of a simple moving average (SMA). An “average age” is the amount of time by which moving averages will tend to lag behind turning points in the original data. The “average age” in this case is (N − 1)/2.

Mathematically, the formulas for MACD and its signal line are the following.

$$\begin{aligned} MACD = EMA(P,N1) - EMA(P,{\text N2}),{\text{ where N1}} \, < \,{\text {N2}} \hfill \\ {\text{Signal}} - MACD = EMA(MACD,N3) \hfill \\ \end{aligned}$$

*P*_*t*_ is the closing price at time t, *N* is the number of days and EMA stands for exponential moving average.

The standard values for “Ns” are 12 days (N1), 26 days (N2) and 9 days (N3) (Colby 2003). This paper also searches for optimal parameter values and then compares results with that from standard parameters.

The buy signal to enter a long position (or to cover a prior short position) is generated when the MACD crosses above its own Signal Line (Bullish signal). On the other hand, the sell signal to enter a short position (or to close a prior long position) is generated when the MACD crosses below its own Signal Line (Bearish signal).

### Directional Movement Indicator (DMI)

The DMI is a filtered momentum or trend-following indicator. Fundamentally, it is a directional movement measure standardized by volatility. The DMI is designed to give buy or sell signal only when a market shows significant trending characteristics to avoid unprofitable trades by following a non-existing trend during a sideways market (Wilder 1978). When a market exhibit no trending behavior, the DMI would tell investors to keep out of the market.

Wilder (1978) also introduces Average Directional Movement Index (ADX) as a measure of trend strength. The buy or sell signals are generated from the DMI only if the ADX indicates that there is a strong trend.

Computationally, both DMI and ADX are calculated in the following steps.

1. Calculate a measure of volatility called True Rang (TR).
  $${\text{TR}} = {\text{Max}}\left[ {|{\text{High}} - {\text{Low}}\left| {, \, } \right|{\text{High}} - {\text{Previous Close}}\left| {, \, } \right|{\text{Low}} - {\text{Previous Close}}|} \right]$$
2. Calculate average true range [ATR(N1)] by summing TR over N1 days. Then, perform a Wilder’s smoothing over TR(N1) by using the following formulas.
  $${\text{First ATR}}\left( {\text{N1}} \right) = {\text{Sum of the first N1 periods of TR}}$$
  $${\text{Subsequent ATR}}\left( {\text{N1}} \right) = {\text{Prior ATR}}\left( {\text{N1}} \right){-}\left[ {{\text{Prior ATR}}\left( {\text{N1}} \right)/{\text{N1}}} \right] + {\text{Current TR}}$$
3. Calculate UpMove and DownMove with the following formulas.
  $${\text{UpMove}} = {\text{today}}'{\text{s Hight}}{-}{\text{yesterday}}'{\text{s High}}$$
  $${\text{DownMove}} = {\text{yesterday}}'{\text{s Low}}{-}{\text{today}}'{\text{s Low}}$$
4. Calculate directional movement (DM) with the following formulas.
  $$\begin{aligned} {\text{If UpMove}} > 0{\text{ and UpMove}} > {\text{DownMove}}, \hfill \\ \;{\text{then }} + {\text{DM}} = {\text{UpMove}},{\text{ Else }} + {\text{DM}} = 0. \hfill \\ \end{aligned}$$
  $$\begin{aligned} {\text{If DownMove}} > 0{\text{ and DownMove}} > {\text{UpMove}}. \hfill \\ {\text{then }} - {\text{DM}} = {\text{DownMove}},{\text{ Else }} - {\text{DM}} = 0. \hfill \\ \end{aligned}$$
5. Calculate DM(N1) by summing DM over N1 days. Then, perform a Wilder’s smoothing over DM (N1) by using the following formulas.
  $${\text{First DM}}\left( {\text{N1}} \right) = {\text{Sum of the first N1 periods of DM}}$$
  $${\text{Subsequent DM}}\left( {\text{N1}} \right) = {\text{Prior DM }}\left( {\text{N1}} \right){-}\left[ {{\text{Prior DM}}\left( {\text{N1}} \right)/{\text{N1}}} \right] + {\text{Current DM}}.$$
6. Calculate Directional Movement Indicator (DMI), which is a standardized DM over a period of N1 days. It is standardized by a volatility measure called ATR(N1).
  Positive Directional Indicator (PDI)
  $${\text{PDI}}\left( {\text{N1}} \right) = \left[ { + {\text{DM}}\left( {\text{N1}} \right)} \right]/\left[ {{\text{ATR}}\left( {\text{N1}} \right)} \right] \times 100$$
  Minus Directional Indicator (MDI)
  $${\text{MDI}}\left( {\text{N1}} \right) = \left[ { - {\text{DM}}\left( {\text{N1}} \right)} \right]/\left[ {{\text{ATR}}\left( {\text{N1}} \right)} \right] \, \times { 1}00.$$
7. Calculate Directional Movement Index (DX). It measures the trend strength of each day based on a price pattern over previous N1 days. Unlike DMI, it does not indicate any price movement directions.
  $${\text{DX}}\left( {\text{N1}} \right) = \frac{{\left| {{\text{PDI}}\left( {\text{N1}} \right)){-}{\text{MMI}}\left( {\text{N1}} \right))} \right|}}{{\left( {{\text{PDI}}\left( {\text{N1}} \right) + {\text{MDI}}\left( {\text{N1}} \right)} \right)}} \times 100$$
8. Calculate average Directional Movement over N1 days [ADX(N1)] by performing a Wilder’s smoothing over DX with the following formulas.
  $${\text{First ADX}}\left( {\text{N1}} \right) = {\text{Simple average of first N1 periods of DX}}\left( {\text{N1}} \right).$$
  $${\text{Subsequent ADX}}\left( {\text{N1}} \right) = \left[ {{\text{Previous ADX}}\left( {\text{N1}} \right)} \right]{\text{x}}\left( {{\text{N1}} - 1} \right) + {\text{Current DX}}\left( {\text{N1}} \right)/{\text{N1}}.$$
9. Calculate average directional movement rating (ADXR) as the simple average of today’s ADX and ADX of N1 days ago.

The ADX does not indicate trend direction or momentum. It only measures trend strength. It is a lagging indicator in a sense that a trend must have established firmly before the ADX will generate a signal that a trend is under way. The ADX varies between 0 and 100. Generally, ADX readings below 20 indicate trend weakness and readings above 40 and 50 indicate a strong trend and an extremely strong trend, respectively. However, one major problem with the ADX is that it is too volatile. The ADXR improves over the ADX on this respect by using the average instead of a single number. In general, ADXR less than 20 indicates a trendless market, while ADXR greater than 25 indicates a trending market.

The standard value for “N1” is 14 days (Colby 2003). This paper also searches for optimal parameter values and then compares results with that from standard parameters.

The buy signal to enter long position is generated when PDI(N1) > MDI(N1) and ADXR > 25 and the position is reversed when PDI(N1) < MDI(N1) or ADXR < 25. On the other hand, the sell signal to enter short position is generated when MDI(N1) > PDI(N1) and ADXR > 25 and the position is reversed when MDI(N1) < PDI(N1) or ADXR < 25.

### On Balance Volume (OBV)

The OBV is a volume-based indicator that relates volume to price change. Basically, it is a running total of volume. If a closing price today is higher (lower) than a closing price yesterday, then the entire today’s volume will be added (deducted) to (from) the previous day OBV to get today OBV. It does not matter how much the price changes. Only the direction of price change matters.

The underlying assumption is that OBV changes precede price changes. The reason is that smart money (investment made by well-informed and sophisticated investors) are flowing into the stock, reflecting in a rising OBV. When the public starts to follow, both the stock price and OBV will surge even more.

The buy signal to enter long position (or to cover prior short position) is generated when the OBV line crosses above its own N1-day EMA (Bullish signal). On the other hand, the sell signal to enter short position (or to close prior long position) is generated when the OBV line crosses below its own N1-day EMA (Bearish signal).

The standard value for “N1” is 3 days (Colby 2003). This paper also searches for optimal parameter values and then compares results with that from standard parameters.

## Methodology

This section is separated into four parts. The first part discusses measures of risk that we use to evaluate each trading system. The second part explains logics and interpretations of each performance measure. The third part provides statistical methods. The last part discusses the optimization of technical trading rule parameters.

### Risk measures

Risk measures include the standard deviation of daily returns and the “Highest Open Drawdown” (HOD), which is the maximum distance the equity line fell below the initial investment during the back-testing simulation.

### Performance measures

This paper reports popular performance measures among technical traders. Though these measures are rarely used in academic research, they are intuitive and widely monitored by actual traders (MetaStock Professional: User’s Manual 2009).

The performance evaluation of each trading rule is based on the following measures.

#### Performance and annualized performance

A “Performance” number is a percentage measure of how much net profit or loss the trading rule generated based on initial equity at the end of the simulation. An “Annualized Performance” calculates a performance over a year. It equals to a performance multiplied by 365 and divided by the number of days in the simulation. The above formula does not take compounding into account.

The number of days used in the formula is “365”, the number of calendar days in a year, as customary in annualizing return (How to Calculate Annualized Returns 2015) instead of the number of trading days in a year, which is used mostly to annualize volatility.

#### Buy and hold index

This index shows the trading system’s performance, as defined above, when compared to a Buy-and-Hold (BH) strategy’s performance. For example, a value of “10” means that the net profit generated were 10 % larger than that of a BH strategy. A positive number does not mean that a trading strategy generates a positive net profit but simply means that it provides a better return than a BH strategy. Similarly, a negative number does not necessary mean that a trading strategy generates losses but simply means that a simple BH strategy would give a better return.

#### Profit and loss index

This index compares the amount of “Net Profit” (Trade Profit − Trade Loss) to the amount of winning or losing trades. It ranges from −100 (worst) to +100 (best). Mathematically, it is defined by the following equation.

$${\text{Profit and loss index}} = \frac{\text{Net Profit}}{\text{Max(Trade Profit, Trade Loss)}} \times 100$$

A positive index number, say 60, reveals that overall a trading strategy generates a positive net profit. However, it is not always profitable as it incurs losses some of the time. The amounts of loss is 40 % of the total profit it generates, resulting in the net profit of only 60 % of the total profit. The index with a value of “100” mean that a trading strategy generates only profits and never losses. A negative index number has the opposite analogous interpretation.

#### Reward and risk index

This index compares a trading system’s reward to its risk. In this case, a reward is defined as a “Net Profit” (Trade Profit − Trade Loss) from a trading system. The risk is defined as a possible change, both positive and negative, in the equity value from an initial investment. The logic behind is analogous to a standard deviation of returns, which measures the differences of realized returns from the expected return without considering whether they are positive or negative.

A positive change in equity value is measured by a positive net profit from a trading system. A negative change is measured by the HOD, which can be interpreted as the largest possible loss from a trading system during its simulation. As a result, the risk measure is just the summation of a positive net profit and the HOD.

The index is the ratio between the reward and its risk. It ranges from −100 (riskiest) to +100 (safest). Mathematically, it is defined by the following equation.

$${\text{Reward and risk index}} = \frac{\text{Net Profit}}{{ [ {\text{Max(Net Profit, 0) + HOD]}}}} \times 100$$

A positive index number, say 20, reveals that overall a trading strategy generates a positive net profit. The return is 20 % of the amount of risk as measured by a possible change, both positive and negative, in the equity value from an initial investment. The index with a value of “100” mean that a trading strategy generates a positive net profit and there is never a principal loss during a simulation.

A negative index number, say −20, reveals that overall a trading strategy generates a loss. However, the actual loss is only 20 % of the maximum possible loss (HOD) during a simulation. The index with a value of “−100” means that a trading strategy incurs the maximum possible loss (HOD).

#### Trade efficiency

A “Trade Efficiency for long only strategy” is calculated in the following way.

$${\text{Trade Efficiency for long only strategy = }}\frac{{\left( {{\text{Exit price }} - {\text{ Entry price}}} \right)}}{{\left( {{\text{Highest price }} - {\text{ Lowest price}}} \right)}}$$

A “Trade Efficiency for short only strategy” is calculated in the following way.

$${\text{Trade Efficiency for short only strategy = }}\frac{{\left( {{\text{Entry price }} - {\text{ Exit price}}} \right)}}{{\left( {{\text{Highest price }} - {\text{ Lowest price}}} \right)}}$$

The highest (lowest) price is the maximum (minimum) close price when the trading position is still open. It ranges from −100 (trading at worst prices) to +100 (trading at best prices). The reported numbers are averages over the number of trades.

Intuitively, the trade efficiency is the average percent of the potential profit the trading rules realized. If by using the technical trading system, a trader could buy at a relatively low (high) price and sell at a relative high (low) price, then the trade efficiency number would be high (low). As such, the trade efficiency can also be interpreted as the measure of market timing ability.

A negative number means a loss from that particular trade. It is noteworthy that this number can be negative and yet the trading system generates a positive net profit. The reason is that the number is the average over the number of trades and thus it is possible that the profits from a fraction of trades can more than compensate the losses from unprofitable ones.

#### Ratio of average profit (from profitable trades) over average loss (from unprofitable trades)

This number is the ratio of an average profit from profitable trades over an average loss from unprofitable ones. A good trading system would let the profit run while cutting losses quickly, resulting in a high ratio.

#### Percentage of profitable trade

This number gives us the proportion of profitable trades. One minus this number will give the proportion of unprofitable trades. A high number would indicate that the trading system has a high chance of correctly predicting subsequent price changes.

### Testing statistics

First, we calculate continuous-compounding daily returns from closing prices of the stock indices [r_t_ = ln(P_t_/P_t−1_)]. The technical indicators would then provide buy or sell signals. When the buy (sell) signal is under test, the chosen daily returns would be all daily returns after the buy (sell) signal was generated up to the next sell (buy) signal. Let define “Φ” to be the union of all disjoint intervals generated by the buy (sell) signals and let “n” be the number of daily returns in the set “Φ”. Then, the average return of the tested strategy is calculated by the following equation.

$$\bar{r} = \frac{{\sum\limits_{i \in \varPhi } {r_{i} } }}{n},{\text{ where }}\bar{r} \sim N\left(\mu ,\frac{{\sigma^{2} }}{n}\right)$$

Let μ_buy_ and μ_sell_ be the population means of daily returns generated by buy and sell signals, respectively. Also, let σ_buy_ and σ_sell_ be the standard deviations of daily returns generated by buy and sell signals, respectively. We would expect that an average return is positive for a buy signal and negative for a sell signal. So, we test the following one-tailed hypotheses: H_0_: μ_buy_ = 0 vs H_1_: μ_buy_ > 0 and H_0_: μ_sell_ = 0 vs H_1_: μ_sell_ < 0 using the following test statistic.

$$\begin{aligned} Z_{buy} = \frac{{\bar{r}_{buy} }}{\left( S_{buy} / \sqrt{n_{buy}}\right)} , \quad S_{buy} = \sqrt {\frac{{\sum\limits_{{i \in \varPhi_{buy} }}^{{}} {(r_{i} - \bar{r}_{buy} )^{2} } }}{{(n_{buy} - 1)}}} \\ \, Z_{sell} = \frac{{\bar{r}_{sell} }}{ \left({S_{sell}} / \sqrt {n_{sell}} \right)}, \quad S_{sell} = \sqrt {\frac{{\sum\limits_{{i \in \varPhi_{sell} }}^{{}} {(r_{i} - \bar{r}_{sell} )^{2} } }}{{(n_{sell} - 1)}}} \end{aligned}$$

n_buy_ is the number of days the long (buy) position is held, n_sell_ is the number of days the short (sell) position is held,

To test the joint effect of buy and sell signals, the hypothesis H_0_: μ_buy_ − μ_sell_ = 0 vs H_1_: μ_buy_ − μ_sell_ > 0 is also tested using the following statistic.

$$Z_{buy - sell} = \frac{{(\bar{r}_{buy} - \bar{r}_{sell} )}}{{\left[ {S.\left( {\frac{1}{{\sqrt {n_{buy} } }} + \frac{1}{{\sqrt {n_{sell} } }}} \right)} \right]}}, \, S = \sqrt {\frac{{\sum\limits_{{i \in \varPhi_{{buy{\text{ or }}sell}} }}^{{}} {(r_{i} - \bar{r}_{{buy{\text{ or }}sell}} )^{2} } }}{{(n_{buy} + n_{sell} - 1)}}}$$

We assume that the standard deviations of daily returns are the same for those generated by buying signals and by selling signals. Therefore, we use the pooled estimator “S”, the standard error of daily returns estimated from the entire sample, to estimate both σ_buy_ and σ_sell_.

For one-tailed test, the significant level (α) is set at 5 and 1 % and hence, the critical Z values are 1.645 and 2.33, respectively.

So far, we have not considered transaction costs yet. To investigate the profitability of each trading rule after transaction cost, we compute break-even transaction costs to be compared with actual transaction costs. According to Bessembinder and Chan (1998), the additional return (π) generated by technical trading rules relative to a buy-and-hold strategy is given as follows.

$$\varPi = \sum\limits_{i = 1}^{{n_{buy} }} {r_{i} } - \sum\limits_{j = 1}^{{n_{sell} }} {r_{j} }$$

n_buy_ is the number of days the long (buy) position is held, n_sell_ is the number of days the short (sell) position is held, r_i_ is the return of the long (buy) position on day “i”, and r_j_ is the return of the short (sell) position on day “j”.

If we divide the additional return (π) by the numbers of buy and sell signals, this will give us the average additional return per signal or, in other words, the round-trip breakeven cost (C) (Bessembinder and Chan 1998).

$$C = \frac{\varPi }{{(s_{buy} + s_{sell} )}}$$

*s*_buy_ is the number of buy signals generated, *s*_sell_ is the number of sells signals generated.

To be profitable, the breakeven cost (C) or the average additional return per signal must be greater than a round-trip transaction cost.

### Optimization of technical trading rule parameters

Each indicator is characterized by a number of parameters called “Ns”, i.e. N1, N2, N3 and so forth. The “standard” values for these parameters are the most popular numbers used by technical traders as reported in Colby (2003). Standard values are usually the numbers that the creators of a technical indicator proposed. Normally, the numbers generated good profits at a time and a place of its creation. As such, there is noting that guarantee the standard values would generate profits at other times or in other markets. Therefore, it is important that traders optimize over these parameters to improve the trading rule’s performance.

In this paper, the “optimal” values for these parameters are the ones that maximize net profits from the trading strategy based on that particular indicator over a sample period. The optimization is done via the grid-search method.

## Data

Our data cover a period of 14 years from January 2000 to December 2013. The instruments investigated are five Southeast Asian stock market indices: SET index (Thailand), FTSE Bursa Malaysia KLC index (Malaysia), FTSE Straits Times index (Singapore), JSX Composite index (Indonesia), and PSE composite index (the Philippines).

We get estimated round-trip transaction costs from the World Stock Exchange (http://www.cftech.com/BrainBank/FINANCE/WorldStockExchange.html). The estimated round-trip transaction costs (including both buying and selling stocks) for Thai, Malaysian, Singaporean, Indonesian, and Philippine stock markets are 0.5, 1.1, 1.133, 1.3 and 1.5 % of transaction value, respectively.

**Fig. 1:**
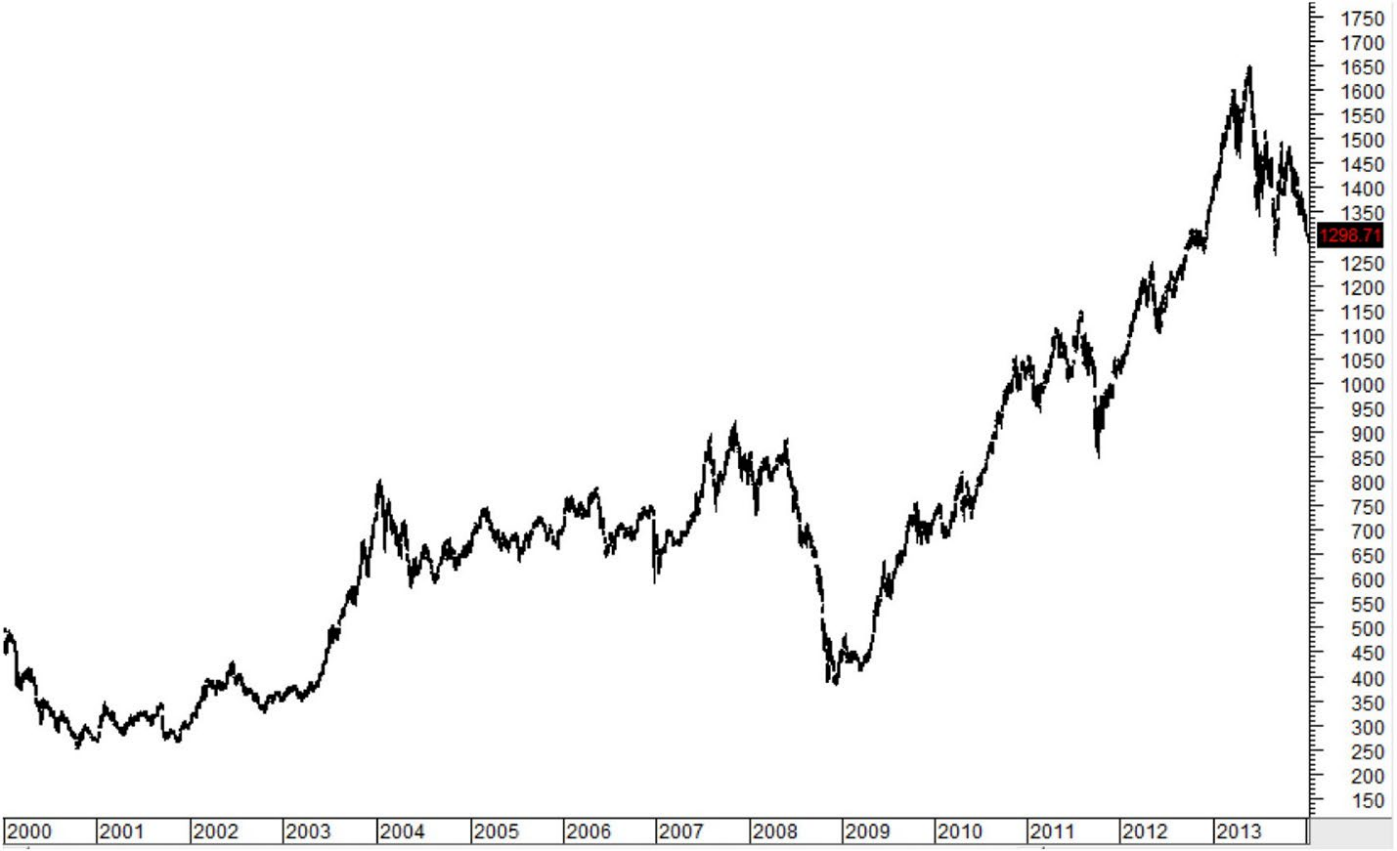
SET index (Thailand) from January 2000 to December 2013

**Fig. 2:**
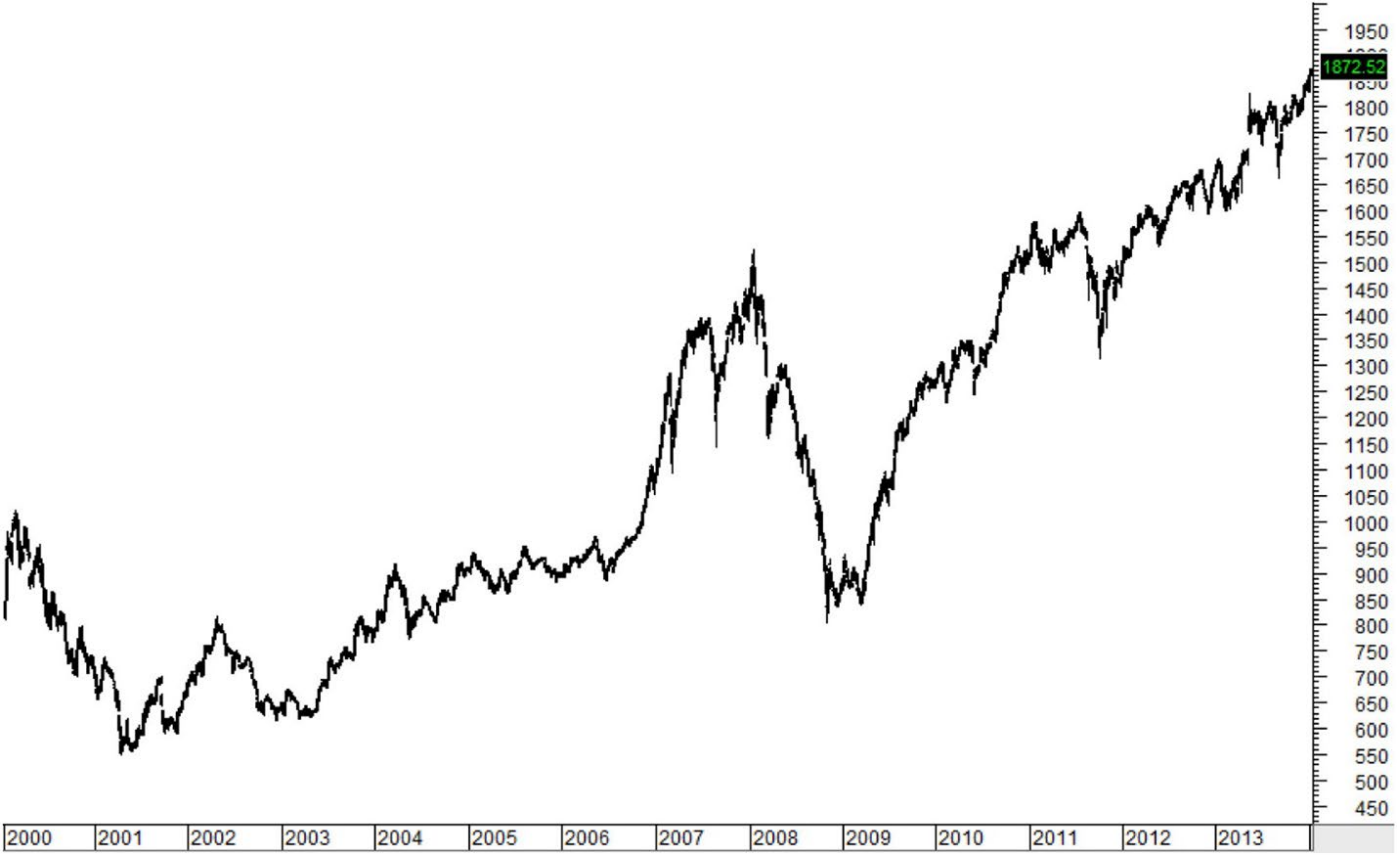
FTSE Bursa Malaysia KLC index (Malaysia) from January 2000 to December 2013

**Fig. 3:**
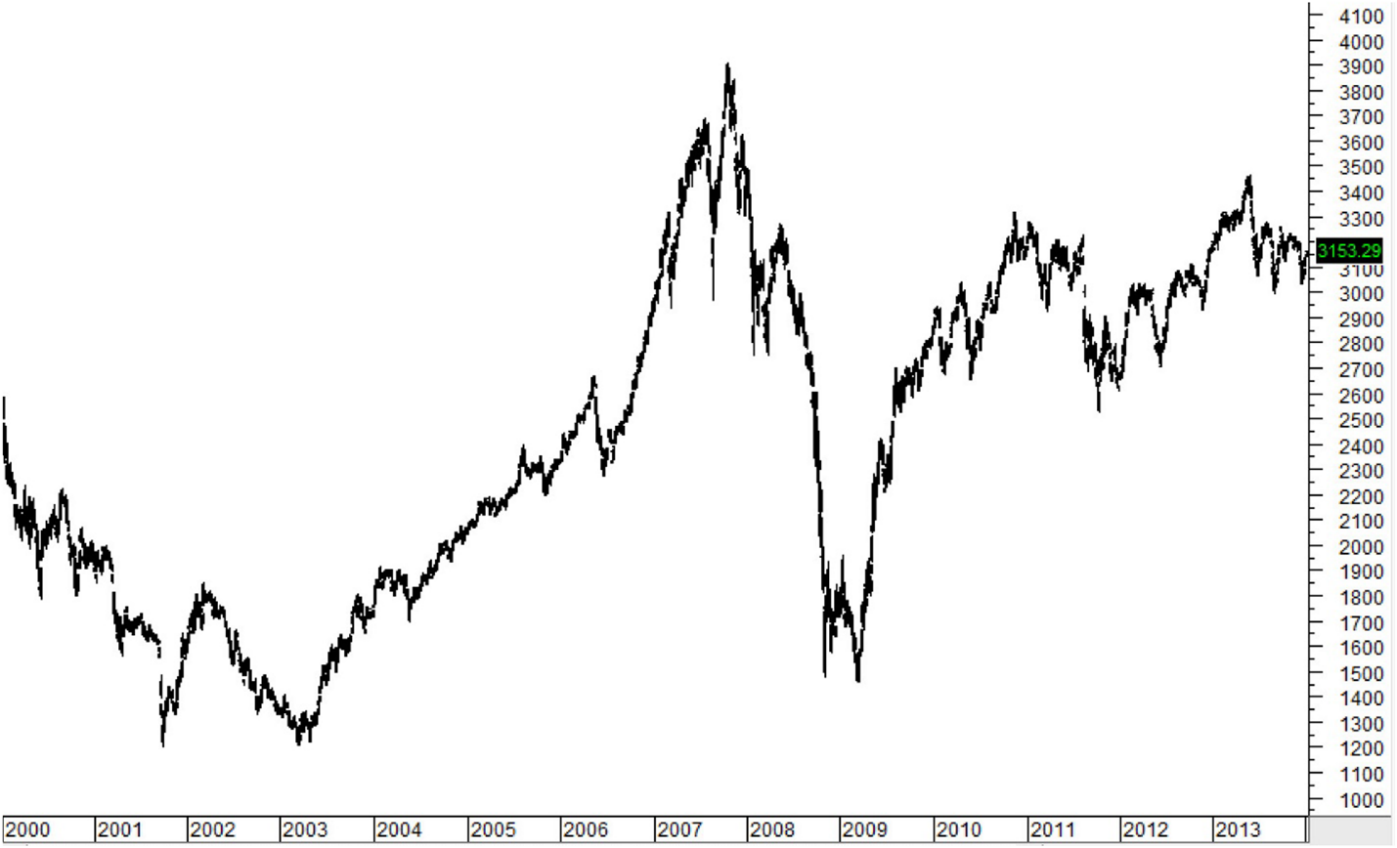
FTSE Straits Times index (Singapore) from January 2000 to December 2013

**Fig. 4:**
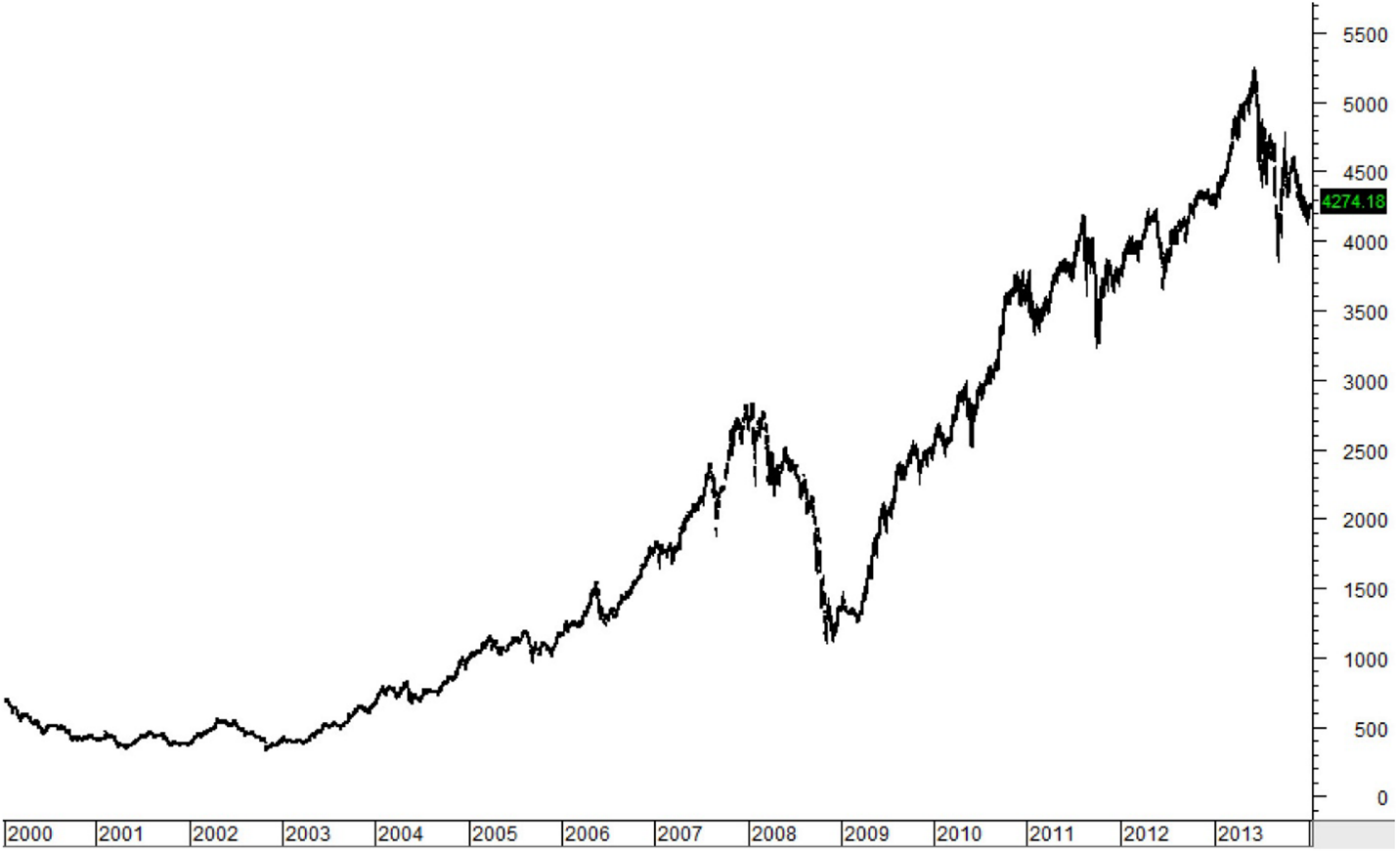
JSX Composite index (Indonesia) from January 2000 to December 2013

**Fig. 5:**
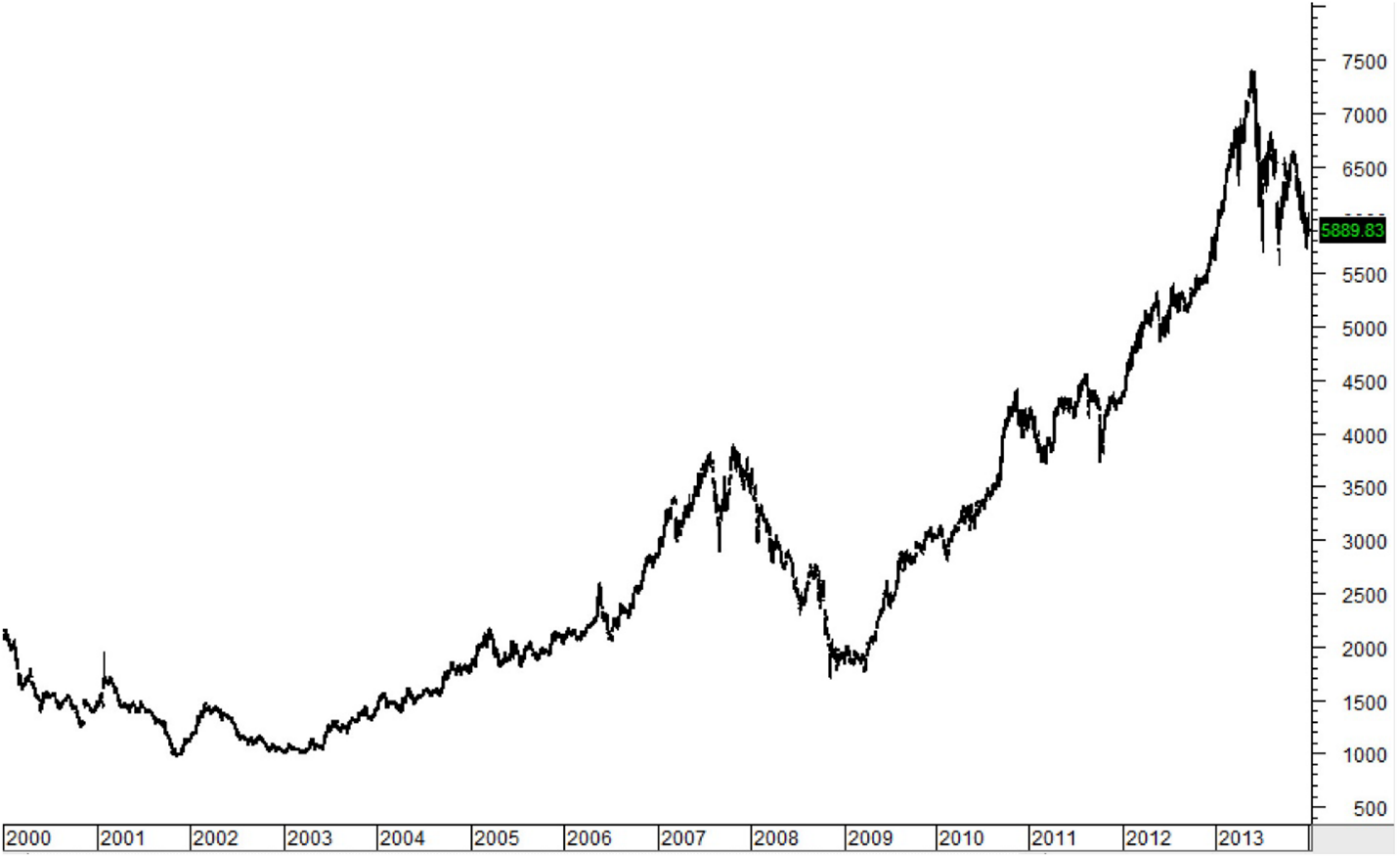
PSE composite index (the Philippines) from January 2000 to December 2013

Figures 1, 2, 3, 4, 5 plot close prices of the SET index (Thailand), FTSE Bursa Malaysia KLC index (Malaysia), FTSE Straits Times index (Singapore), JSX composite index (Indonesia) and PSE composite index (the Philippines) during these 14 years, respectively. All indices had strong uptrends after 2003. Then, they had big drops in 2008 and 2009 due to the Hamburger financial crisis in the US. After that, they recovered and resumed strong uptrends. Most indices (except KLC index) fell and remained in sideway at the latter half of 2013.

Table 1 presents summary statistics. A daily return is calculated as the natural log-difference of an index. The average daily returns are all positive though small, particularly when compared to the standard deviation. The returns are skewed to the left (negative returns). The excess kurtoses indicate that daily return distributions are leptokurtic and have much thicker tails compared to the normal distribution.

**Table 1:** Descriptive statistics of Southeast Asian stock index returns

Statistics	Thailand	Malaysia	Singapore	Indonesia	The Philippines
Observations	3430	3447	3518	3410	3441
Average daily return	0.05 %	0.03 %	0.01 %	0.07 %	0.20 %
Standard deviation of daily return	1.24 %	0.72 %	1.04 %	1.25 %	1.15 %
Maximum	10.58 %	4.17 %	7.53 %	7.62 %	7.06 %
Median	0.04 %	0.03 %	0.03 %	0.09 %	0.02 %
Minimum	−16.06 %	−6.34 %	−8.70 %	−10.95 %	−8.70 %
Skewness	−0.69	−0.37	−0.45	−0.48	−0.34
Excess Kurtosis	13.31	6.23	7.22	6.42	4.85

## Empirical results

### Performances of each trading strategy

Table 2, 3, 4, 5, 6 present performance measures of trading strategies in each market. Consistently, annualized return performances of long-only strategies are higher than those of short-only strategies except for MACD in the Malaysian and the Philippine stock markets. Almost all short-only technical trading strategies perform worse than a Buy-and-Hold (BH). This fact partly reflects general uptrends of the markets during the testing period. This result is to be expected as even a good short-only strategy could not beat a BH strategy in an uptrend market.

**Table 2:** Results of technical trading rules when applied to the SET index (Thailand)

Trading rule results	RSI	Stochastic		MACD	DMI	OBV	Buy and hold (BH)
		STOCH	STOCH-D				
Long strategy							
Performance	7.39 %	2.62 %	382.46 %	400.75 %	176.71 %	60.57 %	161.86 %
Annualized performance	0.53 %	0.19 %	27.34 %	28.65 %	12.63 %	10.25 %	11.57 %
Highest open drawdown (HOD)	37.84 %	39.03 %	22.64 %	10.44 %	33.67 %	15.98 %	49.71 %
Standard deviation of daily returns	1.22 %	1.42 %	1.19 %	1.03 %	1.09 %	0.95 %	1.24 %
Performance indices							
Buy and hold index	−95.43 %	−98.38 %	136.29 %	147.59 %	9.17 %	1.39 %	0.00 %
Profit/loss index	7.00 %	1.00 %	25.91 %	52.45 %	60.35 %	28.31 %	100.00 %
Reward/risk index	16.33 %	6.29 %	94.41 %	97.46 %	84.00 %	79.13 %	76.50 %
Trade summary							
Total trades	16	192	720	130	60	140	n.a.
Trade efficiency	25.11 %	14.60 %	2.32 %	−1.97 %	−1.89 %	−6.52 %	n.a.
Avg. profit/Avg. loss	0.49	0.59	1.47	2.24	2.52	1.97	n.a.
Profitable trades	69 %	63 %	48 %	48 %	50 %	41 %	n.a.
Short strategy							
Performance	−82.51 %	−75.04 %	24.76 %	145.93 %	3.57 %	−11.65 %	161.86 %
Annualized performance	−5.90 %	−5.36 %	1.77 %	10.43 %	0.20 %	−1.97 %	11.57 %
Highest open drawdown (HOD)	85.32 %	77.11 %	18.83 %	0.74 %	25.11 %	21.09 %	49.71 %
Standard deviation of daily returns	1.24 %	1.06 %	1.28 %	1.49 %	1.52 %	1.48 %	1.24 %
Performance indices							
Buy and hold index	−150.98 %	−146.36 %	−84.70 %	−9.84 %	−97.79 %	−119.50 %	0.00 %
Profit/loss index	−65.54 %	−32.64 %	3.48 %	16.08 %	2.98 %	−7.35 %	100.00 %
Reward/risk index	−96.71 %	−97.31 %	56.80 %	99.50 %	12.45 %	−55.25 %	76.50 %
Trade summary							
Total trades	20	192	722	197	62	141	n.a.
Trade efficiency	−5.38 %	−0.78 %	−9.80 %	−19.81 %	−14.16 %	−21.17 %	n.a.
Avg. profit/avg. loss	0.34	0.61	1.63	3.00	1.43	1.80	n.a.
Profitable trades	50 %	53 %	39 %	28 %	42 %	34 %	n.a.

The data cover from January 2000 to December 2013. Due to a data limitation, results of the OBV trading strategy is based on the sample from February 2008 to December 2013. The Buy and Hold index compares OBV performance with that of the BH strategy over the same period, which is 59.74 %. A “performance” is a percentage measure of how much profit or loss the trading rule generated based on initial equity. An “annualized performance” calculates a performance over a year. It equals to a performance multiplied by 365 and divided by the number of days in the simulation. “Highest Open Drawdown” (HOD) is the maximum distance the equity line fell below the initial investment. A “buy and hold index” shows the percentage of the trading system’s profits when compared to a buy-and-hold strategy’s profits. A “profit and loss index” compares the amount of “Net Profit” (Trade Profit − Trade Loss) to the amount of winning or losing trades. Profit and Loss Index = 100 × (Net Profit)/[Max(Trade Profit, Trade Loss)]. A “reward and risk index” compares risk to reward. In this case, risk is defined as the “Highest Open Drawdown” (HOD) plus positive net profit, whereas reward is defined as the “Net Profit” (Trade Profit − Trade Loss) from the trading system. Reward and Risk index = 100 × (Net Profit)/[Max(Net Profit,0) + HOD]. A “trade efficiency” for long only strategy is calculated as (Exit price − Entry price)/(Highest price − Lowest price). A “trade efficiency” for short only strategy is calculated as (Entry price − Exit price)/(Highest price − Lowest price). An “average profit/average loss” is a ratio of average profit of profitable trades over average loss of unprofitable trades

**Table 3:** Results of technical trading rules when applied to the FTSE Bursa Malaysia KLC index (Malaysia)

Trading rule results	RSI	Stochastic		MACD	DMI	OBV	Buy and hold (BH)
		STOCH	STOCH-D				
Long strategy							
Performance	20.27 %	37.48 %	319.44 %	202.27 %	112.62 %	111.97 %	125.53 %
Annualized performance	1.45 %	2.68 %	22.81 %	14.44 %	8.04 %	18.95 %	8.96 %
Highest open drawdown (HOD)	25.24 %	27.70 %	7.55 %	12.39 %	9.43 %	5.89 %	31.81 %
Standard deviation of daily returns	0.81 %	0.80 %	0.70 %	0.80 %	0.62 %	0.52 %	0.72 %
Performance indices							
Buy and hold index	−83.85 %	−70.14 %	154.47 %	61.13 %	−10.28 %	246.23 %	0.00 %
Profit/loss index	24.19 %	18.54 %	34.82 %	58.04 %	62.19 %	66.69 %	100.00 %
Reward/risk index	44.53 %	57.51 %	97.69 %	94.23 %	92.27 %	95.00 %	79.78 %
Trade summary							
Total trades	22	179	718	109	74	122	n.a.
Trade efficiency	35.63 %	17.39 %	1.24 %	7.33 %	−2.86 %	4.52 %	n.a.
Avg. profit/avg. loss	0.29	0.67	1.89	1.68	2.79	3.21	n.a.
Profitable trades	82 %	65 %	45 %	59 %	49 %	48 %	n.a.
Short strategy							
Performance	−64.99 %	−39.33 %	84.58 %	223.29 %	−8.89 %	49.56 %	125.53 %
Annualized performance	−4.64 %	−2.81 %	6.04 %	15.95 %	−0.63 %	8.39 %	8.96 %
Highest open drawdown (HOD)	66.38 %	41.30 %	4.70 %	0.00 %	8.96 %	0.00 %	31.81 %
Standard deviation of daily returns	0.64 %	0.64 %	0.72 %	0.64 %	0.86 %	0.79 %	0.72 %
Performance indices							
Buy and hold index	−151.77 %	−131.33 %	−32.62 %	77.88 %	−107.08	53.25 %	0.00 %
Profit/loss index	−54.17 %	−23.11 %	14.14 %	25.13 %	−9.19 %	43.23 %	100.00 %
Reward/risk index	−97.90 %	−95.23 %	94.74 %	100.00 %	−99.22 %	100.00 %	79.78 %
Trade summary							
Total trades	26	179	718	204	75	122	n.a.
Trade efficiency	0.32 %	2.05 %	−8.08 %	−19.25 %	−23.24 %	−6.35 %	n.a.
Avg. profit/avg. loss	0.46	0.64	1.71	4.72	1.93	2.54	n.a.
Profitable trades	50 %	55 %	41 %	22 %	32 %	41 %	n.a.

The data cover from January 2000 to December 2013. Due to a data limitation, results of the OBV trading strategy is based on the sample from February 2008 to December 2013. The buy and hold index compares OBV performance with that of the BH strategy over the same period, which is 32.34 %. A “performance” is a percentage measure of how much profit or loss the trading rule generated based on initial equity. An “annualized performance” calculates a performance over a year. It equals to a performance multiplied by 365 and divided by the number of days in the simulation. “Highest Open Drawdown” (HOD) is the maximum distance the equity line fell below the initial investment. A “buy and hold index” shows the percentage of the trading system’s profits when compared to a buy-and-hold strategy’s profits. A “profit and loss index” compares the amount of “Net Profit” (Trade Profit − Trade Loss) to the amount of winning or losing trades. Profit and Loss Index = 100 × (Net Profit)/[Max(Trade Profit, Trade Loss)]. A “reward and risk index” compares risk to reward. In this case, risk is defined as the “Highest Open Drawdown” (HOD) plus positive net profit, whereas reward is defined as the “Net Profit” (Trade Profit − Trade Loss) from the trading system. Reward and Risk Index = 100 × (Net Profit)/[Max(Net Profit,0) + HOD]. A “trade efficiency” for long only strategy is calculated as (Exit price − Entry price)/(Highest price − Lowest price). A “trade efficiency” for short only strategy is calculated as (Entry price − Exit price)/(Highest price − Lowest price). An “average profit/average loss” is a ratio of average profit of profitable trades over average loss of unprofitable trades

**Table 4:** Results of technical trading rules when applied to the FTSE Straits Times index (Singapore)

Trading rule results	RSI	Stochastic		MACD	DMI	OBV	Buy and hold (BH)
		STOCH	STOCH-D				
Long strategy							
Performance	−28.57 %	−55.39 %	−12.73 %	53.52 %	40.07 %	113.21 %	26.57 %
Annualized performance	−2.04 %	−3.96 %	−0.91 %	3.82 %	2.86 %	8.08 %	1.90 %
Highest open drawdown (HOD)	60.14 %	63.53 %	37.12 %	24.65 %	11.42 %	22.44 %	49.13 %
Standard deviation of daily returns	1.23 %	1.15 %	1.04 %	0.91 %	0.94 %	0.92 %	1.04 %
Performance indices							
Buy and hold index	−207.53 %	−308.47 %	−147.91 %	101.43 %	50.81 %	326.08 %	0.00 %
Profit/loss index	−40.84 %	−30.67 %	−2.85 %	23.70 %	39.33 %	26.17 %	100.00 %
Reward/risk index	−47.51 %	−87.19 %	−34.29 %	68.47 %	77.82 %	83.45 %	35.10 %
Trade summary							
Total trades	13	204	784	141	58	361	n.a.
Trade efficiency	19.36 %	5.35 %	−3.94 %	−8.81 %	−7.70 %	−6.10 %	n.a.
Avg. profit/avg. loss	0.51	0.57	1.15	1.82	2.18	1.91	n.a.
Profitable trades	54 %	55 %	46 %	42 %	43 %	42 %	n.a.
Short strategy							
Performance	−68.15 %	−75.65 %	−52.14 %	−56.22 %	−30.59 %	19.96 %	26.57 %
Annualized performance	−4.87 %	−5.40 %	−3.72 %	−4.02 %	−2.18 %	1.43 %	1.90 %
Highest open drawdown (HOD)	71.24 %	77.50 %	53.14 %	71.01 %	37.18 %	22.52 %	49.13 %
Standard deviation of daily returns	0.76 %	0.89 %	1.01 %	1.17 %	1.55 %	1.16 %	1.04 %
Performance indices							
Buy and hold index	−356.49 %	−384.72 %	−296.24 %	−311.59 %	−215.13 %	−24.88 %	0.00 %
Profit/loss index	−75.35 %	−42.25 %	−12.41 %	−21.13 %	−36.02 %	6.61 %	100.00 %
Reward/risk index	−95.67 %	−97.61 %	−98.12 %	−79.17 %	−82.26 %	46.98 %	35.10 %
Trade summary							
Total trades	14	203	785	248	57	361	n.a.
Trade efficiency	−3.54 %	−5.20 %	−11.71 %	−26.96 %	−27.32 %	−13.95 %	n.a.
Avg. profit/Avg. loss	0.33	0.64	1.32	2.83	1.97	2.00	n.a.
Profitable trades	43 %	47 %	40 %	22 %	25 %	35 %	n.a.

The data cover from January 2000 to December 2013. A “performance” is a percentage measure of how much profit or loss the trading rule generated based on initial equity. An “annualized performance” calculates a performance over a year. It equals to a performance multiplied by 365 and divided by the number of days in the simulation. “Highest Open Drawdown” (HOD) is the maximum distance the equity line fell below the initial investment. A “buy and hold index” shows the percentage of the trading system’s profits when compared to a buy-and-hold strategy’s profits. A “profit and loss index” compares the amount of “Net Profit” (Trade Profit − Trade Loss) to the amount of winning or losing trades. Profit and Loss Index = 100 × (Net Profit)/[Max(Trade Profit, Trade Loss)]. A “reward and risk index” compares risk to reward. In this case, risk is defined as the “Highest Open Drawdown” (HOD) plus positive net profit, whereas reward is defined as the “Net Profit” (Trade Profit − Trade Loss) from the trading system. Reward and Risk Index = 100 × (Net Profit)/[Max(Net Profit,0) + HOD]. A “trade efficiency” for long only strategy is calculated as (Exit price − Entry price)/(Highest price − Lowest price). A “trade efficiency” for short only strategy is calculated as (Entry price − Exit price)/(Highest price − Lowest price). An “average profit/average loss” is a ratio of average profit of profitable trades over average loss of unprofitable trades

**Table 5:** Results of technical trading rules when applied to the JSX Composite index (Indonesia)

Trading rule results	RSI	Stochastic		MACD	DMI	OBV	Buy and hold (BH)
		STOCH	STOCH-D				
Long strategy							
Performance	−7.45 %	82.14 %	1,391.21 %	396.61 %	378.26 %	863.60 %	517.05 %
Annualized performance	−0.53 %	5.87 %	99.39 %	28.33 %	27.02 %	61.70 %	36.94 %
Highest open drawdown (HOD)	50.35 %	46.08 %	16.79 %	18.48 %	2.05 %	19.89 %	49.48 %
Standard deviation of daily returns	1.32 %	1.40 %	1.17 %	1.06 %	1.04 %	1.05 %	1.25 %
Performance indices							
Buy and hold index	−101.44 %	−84.11 %	169.07 %	−23.29 %	−26.84 %	67.02 %	0.00 %
Profit/loss index	−7.49 %	27.41 %	35.80 %	44.32 %	72.19 %	38.88 %	100.00 %
Reward/risk index	−14.79 %	64.06 %	98.81 %	95.55 %	99.46 %	97.75 %	91.27 %
Trade summary							
Total trades	15	174	689	130	60	330	n.a.
Trade efficiency	23.26 %	14.88 %	6.81 %	−1.61 %	6.18 %	−1.53 %	n.a.
Avg. profit/avg. loss	0.34	0.78	1.54	2.37	3.60	2.00	n.a.
Profitable trades	73 %	64 %	50 %	43 %	50 %	45 %	n.a.
Short strategy							
Performance	−94.33 %	−83.80 %	62.70 %	−6.85 %	23.21 %	15.96 %	517.05 %
Annualized performance	−6.74 %	−5.99 %	4.48 %	−0.49 %	1.66 %	1.14 %	36.94 %
Highest open drawdown (HOD)	95.19 %	87.45 %	1.64 %	20.36 %	8.50 %	0.00 %	49.48 %
Standard deviation of daily returns	1.17 %	1.13 %	1.30 %	1.51 %	1.66 %	1.55 %	1.25 %
Performance indices							
Buy and hold index	−118.24 %	−116.21 %	−87.87 %	−101.32 %	−95.51 %	−96.91 %	0.00 %
Profit/loss index	−78.32 %	−40.08 %	6.26 %	−1.32 %	17.75 %	4.14 %	100.00 %
Reward/risk index	−99.10 %	−95.82 %	97.45 %	−33.65 %	−73.19 %	100.00 %	91.27 %
Trade summary							
Total trades	21	175	690	241	57	331	n.a.
Trade efficiency	−20.51 %	2.39 %	−11.59 %	−22.60 %	−17.58 %	−17.92 %	n.a.
Avg. profit/avg. loss	0.43	0.44	1.82	3.19	1.93	2.38	n.a.
Profitable trades	33 %	58 %	37 %	24 %	39 %	31 %	n.a.

The data cover from January 2000 to December 2013. A “performance” is a percentage measure of how much profit or loss the trading rule generated based on initial equity. An “annualized performance” calculates a performance over a year. It equals to a performance multiplied by 365 and divided by the number of days in the simulation. “Highest Open Drawdown” (HOD) is the maximum distance the equity line fell below the initial investment. A “buy and hold index” shows the percentage of the trading system’s profits when compared to a buy-and-hold strategy’s profits. A “profit and loss index” compares the amount of “Net Profit” (Trade Profit − Trade Loss) to the amount of winning or losing trades. Profit and Loss Index = 100 × (Net Profit)/[Max(Trade Profit, Trade Loss)]. A “reward and risk index” compares risk to reward. In this case, risk is defined as the “Highest Open Drawdown” (HOD) plus positive net profit, whereas reward is defined as the “Net Profit” (Trade Profit − Loss) from the trading system. Reward and Risk Index = 100 × (Net Profit)/[Max(Net Profit,0) + HOD]. A “trade efficiency” for long only strategy is calculated as (Exit price − Entry price)/(Highest price − Lowest price). A “trade efficiency” for short only strategy is calculated as (Entry price − Exit price)/(Highest price − Lowest price). An “average profit/average loss” is a ratio of average profit of profitable trades over average loss of unprofitable trades

**Table 6:** Results of technical trading rules when applied to the PSE composite index (the Philippines)

Trading rule results	RSI	Stochastic		MACD	DMI	OBV	Buy and hold (BH)
		STOCH	STOCH-D				
Long strategy							
Performance	−40.04 %	147.58 %	1176.80 %	397.85 %	87.21 %	27.02 %	175.49 %
Annualized performance	−2.86 %	10.55 %	84.11 %	28.43 %	6.23 %	13.60 %	12.54 %
Highest open drawdown (HOD)	65.39 %	54.97 %	28.75 %	3.20 %	23.61 %	2.64 %	52.70 %
Standard deviation of daily returns	1.21 %	1.18 %	1.16 %	1.04 %	1.05 %	0.85 %	1.15 %
Performance indices							
Buy and hold index	−122.82 %	−15.90 %	570.58 %	126.71 %	−50.30 %	−13.17 %	0.00 %
Profit/loss index	−51.85 %	37.61 %	45.83 %	55.91 %	49.28 %	39.52 %	100.00 %
Reward/risk index	−61.23 %	72.86 %	97.62 %	99.20 %	78.64 %	91.11 %	76.91 %
Trade summary							
Total trades	13	205	676	118	71	56	n.a.
Trade efficiency	2.93 %	17.17 %	2.35 %	0.39 %	−11.26 %	1.26 %	n.a.
Avg. profit/avg. loss	0.41	0.83	1.98	2.51	2.86	2.05	n.a.
Profitable trades	54 %	66 %	48 %	47 %	41 %	45 %	n.a.
Short strategy							
Performance	−91.59 %	−37.66 %	213.29 %	457.66 %	−17.88 %	−1.74 %	175.49 %
Annualized performance	−6.55 %	−2.69 %	15.24 %	32.71 %	−1.28 %	−0.88 %	12.54 %
Highest open drawdown (HOD)	92.95 %	50.51 %	3.84 %	1.45 %	25.83 %	17.80 %	52.70 %
Standard deviation of daily returns	1.09 %	1.11 %	1.12 %	1.29 %	1.45 %	1.15 %	1.15 %
Performance indices							
Buy and hold index	−152.19 %	−121.46 %	21.54 %	160.79 %	−110.19 %	−105.59 %	0.00 %
Profit/loss index	−94.43 %	−15.00 %	19.44 %	25.81 %	−14.96 %	−5.31 %	100.00 %
Reward/risk index	−98.53 %	−74.56 %	98.23 %	99.68 %	−69.21 %	−9.77 %	76.91 %
Trade summary							
Total trades	16	206	677	183	70	55	n.a.
Trade efficiency	−31.56 %	4.68 %	−5.49 %	−14.95 %	−18.97 %	−18.37 %	n.a.
Avg. profit/avg. loss	0.12	0.66	1.72	3.79	1.53	2.12	n.a.
Profitable trades	31 %	56 %	42 %	26 %	36 %	31 %	n.a.

The data cover from January 2000 to December 2013. Due to a data limitation, results of the OBV trading strategy is based on the sample from January 2012 to December 2013. The Buy and hold index compares OBV performance with that of the BH strategy over the same period, which is 31.12 %. A “performance” is a percentage measure of how much profit or loss the trading rule generated based on initial equity. An “annualized performance” calculates a performance over a year. It equals to a performance multiplied by 365 and divided by the number of days in the simulation. “Highest Open Drawdown” (HOD) is the maximum distance the equity line fell below the initial investment. A “buy and hold index” shows the percentage of the trading system’s profits when compared to a buy-and-hold strategy’s profits. A “profit and loss index” compares the amount of “Net Profit” (Trade Profit − Trade Loss) to the amount of winning or losing trades. Profit and Loss Index = 100 × (Net Profit)/[Max(Trade Profit, Trade Loss)]. A “reward and risk index” compares risk to reward. In this case, risk is defined as the “Highest Open Drawdown” (HOD) plus positive net profit, whereas reward is defined as the “Net Profit” (Trade Profit − Trade Loss) from the trading system. Reward and Risk Index = 100 × (Net Profit)/[Max(Net Profit,0) + HOD]. A “trade efficiency” for long only strategy is calculated as (Exit price − Entry price)/(Highest price − Lowest price). A “trade efficiency” for short only strategy is calculated as (Entry price − Exit price)/(Highest price − Lowest price). An “average profit/average loss” is a ratio of average profit of profitable trades over average loss of unprofitable trades

The performance and annualized performance of each trading strategy are compared to those of a BH strategy. Trading strategies that beat a BH are called profitable strategies and have positive buy and hold indices. On the opposite, trading strategies that are beaten by a BH are called unprofitable strategies and have negative buy and hold indices.

The long-only RSI and STOCH trading strategies always perform far worse than a BH, though some time, they generate absolute positive returns. The buy and hold index of long-only RSI and STOCH trading strategies are normally huge negative. The long-only DMI trading strategy also rarely worked as it beat a BH only in the Thai and Singaporean markets with the buy and hold index of 9.17 % and 50.81 % respectively.

In our sample, there is no single best trading strategy, which always outperforms a BH. The long-only STOCH-D trading strategy came close as it had always beaten a BH except only in the Singaporean market. Except in the Singaporean market, its buy and hold index is always higher than 100 % and reaches the maximum of 570.58 % in the Philippine market. Similarly, MACD had outperformed a BH in every market except in the Indonesian market. Except in the Indonesian market, its buy and hold index is normally higher than 100 % with the minimum of 61.13 % in the Malaysian market. The OBV trading strategy also performed well against a BH except in the Philippine market, but we avoid to draw too much inference from that because of limited samples. Except in the Philippine market, its buy and hold index varies from 1.39 % to 326.08 %.

Interestingly, even for profitable trading strategies such as STOCH-D or MACD long-only trading strategies, the percent of profitable trades over total number of trades is still usually less than 50 %. This means that profitable strategies make money not so much from correctly predicting directions of the market, but from letting the profits to run in profitable trades while minimizing loss in unprofitable ones. This fact is reflected in larger than one ratios of average profit over average loss. In sharp contrast, unprofitable long-only strategies like RSI are profitable more than 50 % of the times and sometime up to 80 %, yet it still gives minuscule annualized returns or even negative ones. The average profits over average losses of RSI are much lower than one. No wonder, they are beaten by a BH.

In terms of market timing ability as measured by trade efficiency, we find that using technical indicators does not help much. The trade efficiency measures are normally low and sometime negative even for profitable strategies. For example, trade efficiency of long-only STOCH-D and MACD strategy is just 2.32 % and −1.97 % respectively in the case of the Thai market. The number clearly shows that a technical trading rule generates less than three percent of the potential profit if traders were to buy at the minimum and sell at the maximum prices.

In terms of trading frequency among long-only profitable strategies, the STOCH-D strategy has a very high trading frequency of more than four rounds per month, whereas the OBV strategy also has a high trading frequency of about two rounds per month. The MACD trading strategy has a relatively low trading frequency of only around 0.6–0.8 rounds per month.

The profit and loss indices of profitable strategies like long STOCH-D, MACD, DMI and OBV vary widely across markets from 23.70 % to 72.19 %. Normally, DMI and MACD would have the highest and second highest indices, respectively. The interpretation is that even profitable strategies do not generate profits in every trade and traders can expect losses at least about thirty percent of the total profits generated.

The reward and risk indices of profitable strategies like long STOCH-D, MACD, DMI and OBV are normally very high. They are normally higher than 90 %. This reflects the fact that profitable strategies tend to have limited risk in terms of the Highest Open Drawdown (HOD).

In terms of risk as measured by the HOD, profitable trading strategies such as STOCH-D, MACD, DMI and OBV always have lower risk than a BH. The main reason is that these strategies have a stop-loss function built-in and avoid entering the market during down trends, unlike a BH strategy that investors always fully invest in the indices. Interestingly, unprofitable trading strategies such as RSI and STOCH sometime are riskier or about as risky as a BH. They are also always riskier than the above profitable strategies.

In terms of risk as measured by standard deviation of daily returns, there are not much differences across trading strategies or even across markets. The average number is just above one percentage point.

In summary, we find that in general long-only strategies performed better than similar short-only strategies. This partly reflects general uptrends during the sample period. The simulation also reveals that trading strategies based on MACD and STOCH-D outperformed a BH in most circumstances, while those based on RSI and STOCH always underperformed. The DMI trading strategy performed well only in two markets and was worse than a BH in the other three. The OBV trading strategy generally performed well against a BH, but we avoid drawing too much conclusion because of limited data. The profitable strategies are also less risky than a BH as they have lower Highest Open Drawdowns (HODs). In contrast, unprofitable trading strategies such as RSI and STOCH are at least as risky as a BH.

### Hypothesis testing

Tables 7, 8, 9, 10, 11 reports formal statistical test results. The null hypothesis is that average daily return of each strategy is zero. The alternative hypotheses are that average daily return is positive (for long-only strategies), negative (for short-only strategies) and positive (for long-and-short strategies). The break even trading costs for each strategy are also reported and compared to the actual round trip trading cost of each market.

**Table 7:** Standard test results from SET index (Thailand)

	Long	Short	Long-short
RSI			
Average daily return of a strategy	0.00 %	0.04 %	−0.04 %
SD of daily return of a strategy	1.22 %	1.24 %	1.23 %
Z statistics	0.05	1.32	−0.64 %
Breakeven trading cost (round trip)	0.23 %	Unprofitable	Unprofitable
Number of signal generated	16	20	36
STOCH			
Average daily return of a strategy	0.00 %	0.04 %	−0.04 %
SD of daily return of a strategy	1.42 %	1.06 %	1.23 %
Z statistics	−0.09	1.58	−0.70
Breakeven trading cost (round trip)	Unprofitable	Unprofitable	Unprofitable
Number of signal generated	192	192	384
STOCH-D			
Average daily return of a strategy	0.08 %	−0.05 %	0.13 %
SD of daily return of a strategy	1.19 %	1.28 %	1.23 %
Z statistics	2.91**	−1.54	2.20*
Breakeven trading cost (round trip)	0.30 %	0.16 %	0.23 %
Number of signal generated	720	721	1441
MACD			
Average daily return of a strategy	0.07 %	−0.07 %	0.14 %
SD of daily return of a strategy	1.03 %	1.49 %	1.25 %
Z statistics	2.87**	−1.75*	2.21*
Breakeven trading cost (round trip)	1.44 %^a^	0.71 %^a^	1.00 %^a^
Number of signal generated	130	197	327
DMI			
Average daily return of a strategy	0.08 %	−0.04 %	0.11 %
SD of daily return of a strategy	1.09 %	1.52 %	1.31 %
Z statistics	2.22*	−0.72	1.32
Breakeven trading cost (round trip)	1.91 %^a^	0.78 %^a^	1.33 %^a^
Number of signal generated	60	62	122
OBV			
Average daily return of a strategy	0.07 %	−0.05 %	0.12 %
SD of daily return of a strategy	0.95 %	1.48 %	1.20 %
Z statistics	2.15*	−0.84	1.34
Breakeven trading cost (round trip)	0.64 %^a^	0.31 %	0.48 %
Number of signal generated	140	141	281

The data cover from January 2000 to December 2013. Due to a data limitation, results of the OBV trading strategy is based on the sample from February 2008 to December 2013

*^,^ ** Mean significance at 5 and 1 %, respectively

^a^Means that the breakeven trading cost (round trip) is higher than the actual round trip trading cost of 0.5 % in the Thai stock market. The alternative hypothesis of the long-only, short-only, and long-and-short strategies are that average daily returns are positive, negative and positive, respectively. For one-tailed test, the significant level (α) is set at 5 and 1 % and hence, the critical Z values are 1.645 and 2.33, respectively

**Table 8:** Standard test results from FTSE Bursa Malaysia KLC index (Malaysia)

	Long	Short	Long–short
RSI			
Average daily return of a strategy	0.01 %	0.02 %	−0.01 %
SD of daily return of a strategy	0.81 %	0.64 %	0.71 %
Z statistics	0.43	1.28	−0.25
Breakeven trading cost (round trip)	0.86 %	Unprofitable	Unprofitable
Number of signal generated	22	26	48
STOCH			
Average daily return of a strategy	0.02 %	0.01 %	0.01 %
SD of daily return of a strategy	0.80 %	0.64 %	0.72 %
Z statistics	0.81	0.72	0.17
Breakeven trading cost (round trip)	0.21 %	Unprofitable	0.03 %
Number of signal generated	179	179	358
STOCH-D			
Average daily return of a strategy	0.07 %	−0.05 %	0.12 %
SD of daily return of a strategy	0.70 %	0.72 %	0.72 %
Z statistics	4.15**	−2.59 %**	3.35**
Breakeven trading cost (round trip)	0.25 %	0.16 %	0.21 %
Number of signal generated	718	718	1,436
MACD			
Average daily return of a strategy	0.02 %	−0.05 %	0.12 %
SD of daily return of a strategy	0.80 %	0.72 %	0.72 %
Z statistics	0.81	−2.59**	3.35**
Breakeven trading cost (round trip)	0.21 %	0.16 %	0.21 %
Number of signal generated	179	718	1436
DMI			
Average daily return of a strategy	0.04 %	−0.01 %	0.05 %
SD of daily return of a strategy	0.62 %	0.86 %	0.74 %
Z statistics	2.42**	−0.19*	1.08
Breakeven trading cost (round trip)	1.03 %	0.10 %	0.57 %
Number of signal generated	74	75	149
OBV			
Average daily return of a strategy	0.06 %	−0.06 %	0.12 %
SD of daily return of a strategy	0.52 %	0.79 %	0.65 %
Z statistics	3.42**	−1.92*	2.53**
Breakeven trading cost (round trip)	0.74 %	0.48 %	0.62 %
Number of signal generated	127	122	249

The data cover from January 2000 to December 2013. Due to a data limitation, results of the OBV trading strategy is based on the sample from February 2008 to December 2013

*^,^ ** Mean significance at 5 and 1 %, respectively

^a^Means that the breakeven trading cost (round trip) is higher than the actual round trip trading cost of 1.1 % in the Malaysian stock market. The alternative hypothesis of the long-only, short-only, and long-and-short strategies are that average daily returns are positive, negative and positive, respectively. For one-tailed test, the significant level (α) is set at 5 and 1 % and hence, the critical Z values are 1.645 and 2.33, respectively

**Table 9:** Standard test results from FTSE Straits Times index (Singapore)

	Long	Short	Long–short
RSI			
Average daily return of a strategy	−0.02 %	0.03 %	−0.05 %
SD of daily return of a strategy	1.23 %	0.76 %	1.03 %
Z statistics	−0.57	1.69	−0.96
Breakeven trading cost (round trip)	Unprofitable	Unprofitable	Unprofitable
Number of signal generated	13	14	27
STOCH			
Average daily return of a strategy	−0.02 %	0.03 %	−0.05 %
SD of daily return of a strategy	1.15 %	0.89 %	1.03 %
Z statistics	−0.61	1.34	−0.92
Breakeven trading cost (round trip)	Unprofitable	Unprofitable	Unprofitable
Number of signal generated	204	203	407
STOCH-D			
Average daily return of a strategy	0.02 %	0.00 %	0.02 %
SD of daily return of a strategy	1.04 %	1.01 %	1.03 %
Z statistics	0.66	−0.19	0.43
Breakeven trading cost (round trip)	0.05 %	0.01 %	0.03 %
Number of signal generated	784	785	1569
MACD			
Average daily return of a strategy	0.03 %	−0.04 %	0.06 %
SD of daily return of a strategy	0.91 %	1.17 %	1.04 %
Z statistics	1.19	−1.18	1.19
Breakeven trading cost (round trip)	0.46 %	0.31 %	0.36 %
Number of signal generated	141	248	389
DMI			
Average daily return of a strategy	0.03 %	0.03 %	−0.01 %
SD of daily return of a strategy	0.94 %	1.55 %	1.25 %
Z statistics	0.82	0.56	−0.08
Breakeven trading cost (round trip)	0.55 %	Unprofitable	Unprofitable
Number of signal generated	58	57	115
OBV			
Average daily return of a strategy	0.03 %	−0.03 %	0.06 %
SD of daily return of a strategy	0.92 %	1.16 %	1.03 %
Z statistics	1.51	−0.95	1.20
Breakeven trading cost (round trip)	0.25 %	0.17 %	0.21 %
Number of signal generated	361	361	722

The data cover from January 2000 to December 2013

*^,^ ** Mean significance at 5 % and 1 %, respectively

^a^ Means that the breakeven trading cost (round trip) is higher than the actual round trip trading cost of 1.133 % in the Singaporean stock market. The alternative hypothesis of the long-only, short-only, and long-and-short strategies are that average daily returns are positive, negative and positive, respectively. For one-tailed test, the significant level (α) is set at 5 % and 1 % and hence, the critical Z values are 1.645 and 2.33, respectively

**Table 10:** Standard test results from JSX Composite index (Indonesia)

	Long	Short	Long–short
RSI			
Average daily return of a strategy	0.00 %	0.07 %	−0.07 %
SD of daily return of a strategy	1.32 %	1.17 %	1.25 %
Z statistics	−0.05	2.48	−1.16
Breakeven trading cost (round trip)	Unprofitable	Unprofitable	Unprofitable
Number of signal generated	15	21	36
STOCH			
Average daily return of a strategy	0.04 %	0.03 %	0.01 %
SD of daily return of a strategy	1.40 %	1.13 %	1.25 %
Z statistics	1.05	1.32	0.09
Breakeven trading cost (round trip)	0.47 %	Unprofitable	Unprofitable
Number of signal generated	174	175	349
STOCH-D			
Average daily return of a strategy	0.14 %	-0.08 %	0.22 %
SD of daily return of a strategy	1.17 %	1.30 %	1.25 %
Z statistics	4.98**	−2.44**	3.61**
Breakeven trading cost (round trip)	0.54 %	0.28 %	0.41 %
Number of signal generated	689	690	1379
MACD			
Average daily return of a strategy	0.05 %	−0.03 %	0.08 %
SD of daily return of a strategy	1.06 %	1.51 %	1.27 %
Z statistics	2.17*	−0.65	1.25
Breakeven trading cost (round trip)	1.12 %	0.21	0.53 %
Number of signal generated	130	241	371
DMI			
Average daily return of a strategy	0.10 %	−0.04 %	0.14 %
SD of daily return of a strategy	1.04 %	1.66 %	1.35 %
Z statistics	3.14 %**	−0.69	1.59
Breakeven trading cost (round trip)	2.69 %^a^	0.84 %	1.79 %^a^
Number of signal generated	60	57	117
OBV			
Average daily return of a strategy	0.07 %	−0.04 %	0.11 %
SD of daily return of a strategy	1.05 %	1.55 %	1.25 %
Z statistics	3.36**	−0.86	1.81*
Breakeven trading cost (round trip)	0.76 %	0.20 %	0.48 %
Number of signal generated	330	331	661

The data cover from January 2000 to December 2013

*^,^ ** mean significance at 5 and 1 %, respectively

^a^Means that the breakeven trading cost (round trip) is higher than the actual round trip trading cost of 1.3 % in the Indonesian stock market. The alternative hypothesis of the long-only, short-only, and long-and-short strategies are that average daily returns are positive, negative and positive, respectively. For one-tailed test, the significant level (α) is set at 5 and 1 % and hence, the critical Z values are 1.645 and 2.33, respectively

**Table 11:** Standard test results from PSE composite index (the Philippines)

	Long	Short	Long–short
RSI			
Average daily return of a strategy	−0.02 %	0.06 %	−0.08 %
SD of daily return of a strategy	1.21 %	1.09 %	1.15 %
Z statistics	−0.69	2.41	−1.47
Breakeven trading cost (round trip)	Unprofitable	Unprofitable	Unprofitable
Number of signal generated	13	16	29
STOCH			
Average daily return of a strategy	0.05 %	−0.01 %	0.06 %
SD of daily return of a strategy	1.18 %	1.11 %	1.14 %
Z statistics	1.69*	−0.23	1.01
Breakeven trading cost (round trip)	0.58 %	0.08 %	0.33 %
Number of signal generated	205	206	411
STOCH-D			
Average daily return of a strategy	0.12 %	−0.09 %	0.20 %
SD of daily return of a strategy	1.16 %	1.12 %	1.14 %
Z statistics	4.28**	−3.14**	3.70**
Breakeven trading cost (round trip)	0.46 %	0.31 %	0.39 %
Number of signal generated	676	677	1353
MACD			
Average daily return of a strategy	0.07 %	−0.08 %	0.14 %
SD of daily return of a strategy	1.04 %	1.29 %	1.16 %
Z statistics	2.70**	−2.27*	2.47**
Breakeven trading cost (round trip)	1.48 %	0.87 %	1.11 %
Number of signal generated	118	183	301
DMI			
Average daily return of a strategy	0.04 %	0.00 %	0.04 %
SD of daily return of a strategy	1.05 %	1.45 %	1.24 %
Z statistics	1.30	0.06	0.48
Breakeven trading cost (round trip)	0.96 %	0.05 %	0.46 %
Number of signal generated	71	70	141
OBV			
Average daily return of a strategy	0.07 %	0.01 %	0.06 %
SD of daily return of a strategy	0.85 %	1.15 %	1.01 %
Z statistics	1.11	0.07	0.42
Breakeven trading cost (round trip)	2.02 %^a^	Unprofitable	0.28 %
Number of signal generated	10	55	65

The data cover from January 2000 to December 2013. Due to a data limitation, results of the OBV trading strategy is based on the sample from January 2012 to December 2013

*^,^ ** Mean significance at 5 and 1 %, respectively

^a^Means that the breakeven trading cost (round trip) is higher than the actual round trip trading cost of 1.5 % in the Philippine stock market. The alternative hypothesis of the long-only, short-only, and long-and-short strategies are that average daily returns are positive, negative and positive, respectively. For one-tailed test, the significant level (α) is set at 5 and 1 % and hence, the critical Z values are 1.645 and 2.33, respectively

The results vary from market to market. The Singaporean market is an extreme case as there is no technical trading strategies studied that generate a significant average daily return. In addition, none have breakeven trading costs higher than the actual one. This implies that seemingly profitable strategies like MACD, DMI and OBV are in fact not profitable at all after transaction costs. Basically, they generate too many trades. Our result is similar to Yu et al. (2013).

On the other hand, the Thai market is the opposite extreme case. The STOCH-D, MACD, DMI and OBV trading strategies all generate significant average daily returns. Only the STOCH-D fails to have a breakeven trading cost higher than the actual one. The MACD, DMI and OBV trading strategies are profitable even after transaction costs.

The Malaysian, Indonesian and the Philippine markets are something in between the above extreme cases. In the Malaysian market, the STOCH-D, MACD, DMI and OBV produce highly significant average daily returns, yet none generate profits after transaction costs. In the Philippine market, only STOCH-D and MACD trading strategies generate highly significant average daily returns. Yet again, both of them do not produce after-transaction cost profits. Only the OBV trading strategy produces an after-transaction cost profit, but the average daily return is not statistically significant. In the Indonesian market, the STOCH-D, DMI and OBV trading strategies produce highly significant average daily returns. Nevertheless, only DMI trading strategy could generate a profit after transaction costs.

To summarize, our statistical test results vary widely across markets. On one hand, no technical trading strategies investigated yield a significant average daily return in the Singaporean market. In addition, none give a net return after transaction costs. On the other hand, four trading strategies (STOCH-D, MACD, DMI and OBV) generate significant average daily returns and three strategies (MACD, DMI, OBV) even give net returns after transaction costs in the Thai market. The results from the Malaysian, Indonesian and the Philippine markets fall between the above extreme. In short, profitable strategies produce significant average daily return, but only DMI generates both a significant return and a profit after transaction costs in the Indonesian market.

### Results of trading rules with optimized parameters

Tables 12, 13, 14, 15, 16 compares results from trading rules with standard parameters and those with optimized parameters. One conclusion is clear from our analysis. There are no universal optimal parameters. The optimized parameters are market specific with different values for different markets. The increases in performance also vary widely among markets and trading strategies from dramatic to little improvements.

**Table 12:** Results from SET index (Thailand) with standard parameters compared to optimized parameters

	RSI		STOCH		STOCH-D		MACD		DMI		OBV		Buy and hold (BH)
	Long	Short	Long	Short	Long	Short	Long	Short	Long	Short	Long	Short	
Standard parameters													
N1	14	14	5	5	5	5	12	12	14	14	3	3	n.a.
N2	n.a.	n.a.	1	1	1	1	26	26	n.a.	n.a.	n.a.	n.a.	n.a.
N3	n.a.	n.a.	n.a.	n.a.	3	3	9	9	n.a.	n.a.	n.a.	n.a.	n.a.
Performance	7.39 %	−82.51 %	2.62 %	−75.04 %	382.46 %	24.76 %	400.75 %	145.93 %	176.71 %	3.57 %	60.57 %	−11.65 %	161.86 %
Annualized performance	0.53 %	−5.90 %	0.19 %	−5.36 %	27.34 %	1.77 %	28.65 %	10.43 %	12.63 %	0.20 %	10.25 %	−1.97 %	11.57 %
Highest open drawdown (HOD)	37.84 %	85.32 %	39.03 %	77.11 %	22.64 %	18.83 %	10.44 %	0.74 %	33.67 %	25.11 %	15.98 %	21.09 %	49.71 %
Buy and hold index	−95.43 %	−150.98 %	−98.38 %	−146.36 %	136.29 %	−84.70 %	147.59 %	−9.84 %	9.17 %	−97.79 %	1.39 %	−119.50 %	0.00 %
Profit/loss index	7.00 %	−65.54 %	1.00 %	−32.64 %	25.91 %	3.48 %	52.45 %	16.08 %	60.35 %	2.98 %	28.31 %	−7.35 %	100.00 %
Reward/risk index	16.33 %	−96.71 %	6.29 %	−97.31 %	94.41 %	56.80 %	97.46 %	99.50 %	84.00 %	12.45 %	79.13 %	−55.25 %	76.50 %
Total trades	16	20	192	192	720	722	130	197	60	62	140	141	n.a.
Trade efficiency	25.11 %	−5.38 %	14.60 %	−0.78 %	2.32 %	−9.80 %	−1.97 %	−19.81 %	−1.89 %	−14.16 %	−6.52 %	−21.17 %	n.a.
Avg. profit/avg. loss	0.49	0.34	0.59	0.61	1.47	1.63	2.24	3.00	2.52	1.43	1.97	1.80	n.a.
Profitable trades	69 %	50 %	63 %	53 %	48 %	39 %	48 %	28 %	50 %	42 %	41 %	34 %	n.a.
Optimal parameters													
N1	12	6	8	5	15	8	10	11	8	5	23	23	n.a.
N2	n.a.	n.a.	5	2	2	2	30	26	n.a.	n.a.	n.a.	n.a.	n.a.
N3	n.a.	n.a.	n.a.	n.a.	5	5	5	5	n.a.	n.a.	n.a.	n.a.	n.a.
Performance	42.33 %	−78.42 %	29.10 %	−74.27 %	638.20 %	81.58 %	630.38 %	423.29 %	287.14 %	51.92 %	113.19 %	14.46 %	161.86 %
Annualized performance	3.03 %	−5.61 %	2.08 %	−5.31 %	45.62 %	5.83 %	45.06 %	30.26 %	20.53 %	3.71 %	19.16 %	2.45 %	11.57 %
Highest open drawdown (HOD)	37.84 %	81.99 %	31.58 %	77.47 %	14.45 %	2.20 %	4.11 %	0.00 %	21.53 %	16.44 %	13.79 %	7.32 %	49.71 %
Buy and hold index	−73.84 %	−148.45 %	−82.02 %	−145.89 %	294.30 %	−49.60 %	289.47 %	161.53 %	77.40 %	−67.92 %	89.47 %	−75.80 %	0.00 %
Profit/loss index	28.91 %	−45.11 %	13.06 %	−39.53 %	36.28 %	9.94 %	50.83 %	20.95 %	70.64 %	20.04 %	49.76 %	12.94 %	100.00 %
Reward/risk index	52.80 %	−95.64 %	47.95 %	−95.87 %	97.79 %	97.38 %	99.35 %	100.00 %	93.03 %	75.95 %	89.14 %	66.40 %	76.50 %
Total trades	22	61	64	142	374	394	164	214	87	139	81	82	n.a.
Trade efficiency	34.66 %	2.81 %	17.17 %	2.25 %	4.89 %	−11.29 %	2.08 %	−17.10 %	1.53 %	−13.07 %	−9.54 %	−19.29 %	n.a.
Avg. profit/avg. loss	0.31	0.44	0.69	0.48	1.49	1.68	2.14	2.84	3.18	1.97	4.21	3.13	n.a.
Profitable trades	82 %	56 %	63 %	56 %	51 %	40 %	49 %	31 %	52 %	39 %	32 %	27 %	n.a.

The data cover from January 2000 to December 2013. Due to a data limitation, results of the OBV trading strategy is based on the sample from February 2008 to December 2013. A performance of the BH strategy over the same period is 59.74 %. A “performance” is a percentage measure of how much profit or loss the trading rule generated based on initial equity. An “annualized performance” calculates a performance over a year. It equals to a performance multiplied by 365 and divided by the number of days in the simulation. “Highest Open Drawdown” (HOD) is the maximum distance the equity line fell below the initial investment. A “buy and hold index” shows the percentage of the trading system’s profits when compared to a buy-and-hold strategy’s profits. A “profit and loss index” compares the amount of “Net Profit” (Trade Profit − Trade Loss) to the amount of winning or losing trades. Profit and Loss Index = 100 × (Net Profit)/[Max(Trade Profit, Trade Loss)]. A “reward and risk index” compares risk to reward. In this case, risk is defined as the “Highest Open Drawdown” (HOD) plus positive net profit, whereas reward is defined as the “Net Profit” (Trade Profit − Trade Loss) from the trading system. Reward and Risk Index = 100×(Net Profit)/[Max(Net Profit,0) + HOD]. A “trade efficiency” for long only strategy is calculated as (Exit price − Entry price)/(Highest price − Lowest price). A “trade efficiency” for short only strategy is calculated as (Entry price − Exit price)/(Highest price − Lowest price). An “average profit/average loss” is a ratio of average profit of profitable trades over average loss of unprofitable trades. MACD’s parameter 1, 2 and 3 are optimized over 10–15, 20–30 and 5–15, respectively. RSI’s parameter 1 is optimized over 5–25. STO’s parameter 1 and 2 are optimized over 5–15 and 1–5, respectively. STOD’s parameter 1, 2 and 3 are optimized over 5–15, 1–5 and 3–5, respectively

**Table 13:** Results from FTSE Bursa Malaysia KLC index (Malaysia) with standard parameters compared to optimized parameters

	RSI		STOCH		STOCH-D		MACD		DMI		OBV		Buy and hold (BH)
	Long	Short	Long	Short	Long	Short	Long	Short	Long	Short	Long	Short	
Standard parameters													
N1	14	14	5	5	5	5	12	12	14	14	3	3	n.a.
N2	n.a.	n.a.	1	1	1	1	26	26	n.a.	n.a.	n.a.	n.a.	n.a.
N3	n.a.	n.a.	n.a.	n.a.	3	3	9	9	n.a.	n.a.	n.a.	n.a.	n.a.
Performance	20.27 %	−64.99 %	37.48 %	−39.33 %	319.44 %	84.58 %	202.27 %	223.29 %	112.62 %	−8.89 %	111.97 %	49.56 %	125.53 %
Annualized performance	1.45 %	−4.64 %	2.68 %	−2.81 %	22.81 %	6.04 %	14.44 %	15.95 %	8.04 %	−0.63 %	18.95 %	8.39 %	8.96 %
Highest open drawdown (HOD)	25.24 %	66.38 %	27.70 %	41.30 %	7.55 %	4.70 %	12.39 %	0.00 %	9.43 %	8.96 %	5.89 %	0.00 %	31.81 %
Buy and hold index	−83.85 %	−151.77 %	−70.14 %	−131.33 %	154.47 %	−32.62 %	61.13 %	77.88 %	−10.28 %	−107.08	246.23 %	53.25 %	0.00 %
Profit/loss index	24.19 %	−54.17 %	18.54 %	−23.11 %	34.82 %	14.14 %	58.04 %	25.13 %	62.19 %	−9.19 %	66.69 %	43.23 %	100.00 %
Reward/risk index	44.53 %	−97.90 %	57.51 %	−95.23 %	97.69 %	94.74 %	94.23 %	100.00 %	92.27 %	−99.22 %	95.00 %	100.00 %	79.78 %
Total trades	22	26	179	179	718	718	109	204	74	75	122	122	n.a.
Trade efficiency	35.63 %	0.32 %	17.39 %	2.05 %	1.24 %	−8.08 %	7.33 %	−19.25 %	−2.86 %	−23.24 %	4.52 %	−6.35 %	n.a.
Avg. profit/avg. loss	0.29	0.46	0.67	0.64	1.89	1.71	1.68	4.72	2.79	1.93	3.21	2.54	n.a.
Profitable trades	82 %	50 %	65 %	55 %	45 %	41 %	59 %	22 %	49 %	32 %	48 %	41 %	n.a.
Optimal parameters													
N1	25	25	7	5	12	12	10	10	5	5	9	8	n.a.
N2	n.a.	n.a.	2	1	1	1	20	20	n.a.	n.a.	n.a.	n.a.	n.a.
N3	n.a.	n.a.	n.a.	n.a.	3	3	5	5	n.a.	n.a.	n.a.	n.a.	n.a.
Performance	52.87 %	−44.53 %	41.64 %	−39.33 %	634.26 %	226.86 %	372.74 %	563.07 %	148.18 %	16.56 %	118.20 %	55.93 %	125.53 %
Annualized performance	3.78 %	−3.18 %	2.97 %	−2.81 %	45.30 %	16.20 %	26.62 %	40.21 %	10.58 %	1.18 %	20.00 %	9.46 %	8.96 %
Highest open drawdown (HOD)	24.21 %	51.08 %	24.32 %	41.30 %	0.26 %	1.58 %	4.22 %	0.33 %	3.66 %	4.53 %	3.43 %	0.00 %	31.81 %
Buy and hold index	−57.88 %	−135.47 %	−66.83 %	−131.33 %	405.27 %	80.72 %	196.93 %	348.55 %	18.04 %	−86.81 %	265.49 %	72.94 %	0.00 %
Profit/loss index	58.13 %	−49.88 %	23.83 %	−23.11 %	43.49 %	23.95 %	60.92 %	33.86 %	58.66 %	10.65 %	65.96 %	42.07 %	100.00 %
Reward/RISK index	68.59 %	−87.18 %	63.12 %	−95.23 %	99.96 %	99.31 %	98.88 %	99.94 %	97.59 %	78.54 %	97.18 %	100.00 %	79.78 %
Total trades	9	10	109	179	641	641	165	228	156	149	127	139	n.a.
Trade efficiency	44.29 %	−3.79 %	18.47 %	2.05 %	3.33 %	−7.82 %	6.80 %	−12.98 %	0.63 %	−18.02 %	4.18 %	−5.98 %	n.a.
Avg. profit/avg. loss	1.19	0.50	0.70	0.64	1.91	1.90	2.24	3.41	2.75	2.43	3.18	2.34	n.a.
Profitable trades	67 %	50 %	65 %	55 %	48 %	41 %	53 %	31 %	47 %	32 %	48 %	42 %	n.a.

The data cover from January 2000 to December 2013. Due to a data limitation, results of the OBV trading strategy is based on the sample from February 2008 to December 2013. A performance of the BH strategy over the same period is 32.34 %. A “performance” is a percentage measure of how much profit or loss the trading rule generated based on initial equity. An “annualized performance” calculates a performance over a year. It equals to a performance multiplied by 365 and divided by the number of days in the simulation. “Highest Open Drawdown” (HOD) is the maximum distance the equity line fell below the initial investment. A “buy and hold index” shows the percentage of the trading system’s profits when compared to a buy-and-hold strategy’s profits. A “profit and loss index” compares the amount of “Net Profit” (Trade Profit − Trade Loss) to the amount of winning or losing trades. Profit and Loss Index = 100 × (Net Profit)/[Max(Trade Profit, Trade Loss)]. A “reward and risk index” compares risk to reward. In this case, risk is defined as the “Highest Open Drawdown” (HOD) plus positive net profit, whereas reward is defined as the “Net Profit” (Trade Profit − Trade Loss) from the trading system. Reward and Risk Index = 100 × (Net Profit)/[Max(Net Profit,0) + HOD]. A “trade efficiency” for long only strategy is calculated as (Exit price − price)/(Highest price − Lowest price). A “trade efficiency” for short only strategy is calculated as (Entry price − Exit price)/(Highest price − Lowest price). An “average profit/average loss” is a ratio of average profit of profitable trades over average loss of unprofitable trades. MACD’s parameter 1, 2 and 3 are optimized over 10–15, 20–30 and 5–15, respectively. RSI’s parameter 1 is optimized over 5–25. STO’s parameter 1 and 2 are optimized over 5–15 and 1–5, respectively. STOD’s parameter 1, 2 and 3 are optimized over 5–15, 1–5 and 3–5, respectively

**Table 14:** Results from FTSE Straits Times index (Singapore) with standard parameters compared to optimized parameters

	RSI		STOCH		STOCH-D		MACD		DMI		OBV		Buy and hold (BH)
	Long	Short	Long	Short	Long	Short	Long	Short	Long	Short	Long	Short	
Standard parameters													
N1	14	14	5	5	5	5	12	12	14	14	3	3	n.a.
N2	n.a.	n.a.	1	1	1	1	26	26	n.a.	n.a.	n.a.	n.a.	n.a.
N3	n.a.	n.a.	n.a.	n.a.	3	3	9	9	n.a.	n.a.	n.a.	n.a.	n.a.
Performance	−28.57 %	−68.15 %	−55.39 %	−75.65 %	−12.73 %	−52.14 %	53.52 %	−56.22 %	40.07 %	−30.59 %	113.21 %	19.96 %	26.57 %
Annualized performance	−2.04 %	−4.87 %	−3.96 %	−5.40 %	−0.91 %	−3.72 %	3.82 %	−4.02 %	2.86 %	−2.18 %	8.08 %	1.43 %	1.90 %
Highest open drawdown (HOD)	60.14 %	71.24 %	63.53 %	77.50 %	37.12 %	53.14 %	24.65 %	71.01 %	11.42 %	37.18 %	22.44 %	22.52 %	49.13 %
Buy and hold index	−207.53 %	−356.49 %	−308.47 %	−384.72 %	−147.91 %	−296.24 %	101.43 %	−311.59 %	50.81 %	−215.13 %	326.08 %	−24.88 %	0.00 %
Profit/loss index	−40.84 %	−75.35 %	−30.67 %	−42.25 %	−2.85 %	−12.41 %	23.70 %	−21.13 %	39.33 %	−36.02 %	26.17 %	6.61 %	100.00 %
Reward/risk index	−47.51 %	−95.67 %	−87.19 %	−97.61 %	−34.29 %	−98.12 %	68.47 %	−79.17 %	77.82 %	−82.26 %	83.45 %	46.98 %	35.10 %
Total trades	13	14	204	203	784	785	141	248	58	57	361	361	n.a.
Trade efficiency	19.36 %	−3.54 %	5.35 %	−5.20 %	−3.94 %	−11.71 %	−8.81 %	−26.96 %	−7.70 %	−27.32 %	−6.10 %	−13.95 %	n.a.
Avg. PROFIT/Avg. loss	0.51	0.33	0.57	0.64	1.15	1.32	1.82	2.83	2.18	1.97	1.91	2.00	n.a.
Profitable trades	54 %	43 %	55 %	47 %	46 %	40 %	42 %	22 %	43 %	25 %	42 %	35 %	n.a.
Optimal parameters													
N1	8	8	7	7	7	7	15	15	10	10	6	6	n.a.
N2	n.a.	n.a.	2	2	5	5	30	30	n.a.	n.a.	n.a.	n.a.	n.a.
N3	n.a.	n.a.	n.a.	n.a.	5	5	15	15	n.a.	n.a.	n.a.	n.a.	n.a.
Performance	36.43 %	−40.90 %	55.12 %	−13.44 %	153.52 %	35.13 %	95.91 %	−18.82 %	62.12 %	−18.47 %	130.51 %	29.03 %	26.57 %
Annualized performance	2.60 %	−2.92 %	3.94 %	−0.96 %	10.96 %	2.51 %	6.85 %	−1.34 %	4.44 %	−1.32 %	9.32 %	2.07 %	1.90 %
Highest open drawdown (HOD)	22.31 %	57.36 %	28.31 %	31.72 %	16.02 %	1.60 %	20.33 %	62.64 %	12.99 %	29.13 %	27.02 %	16.00 %	49.13 %
Buy and hold index	37.11 %	−253.93 %	107.45 %	−150.58 %	477.79 %	32.22 %	260.97 %	−170.83 %	133.80 %	−169.51 %	391.19 %	9.26 %	0.00 %
Profit/loss index	26.07 %	−21.20 %	21.27 %	−6.64 %	26.23 %	8.72 %	37.75 %	−6.81 %	47.79 %	−22.42 %	24.57 %	7.17 %	100.00 %
Reward/risk index	62.02 %	−71.31 %	66.07 %	−42.37 %	90.55 %	95.63 %	82.51 %	−30.04 %	82.71 %	−63.38 %	82.85 %	64.48 %	35.10 %
Total trades	40	41	130	129	282	281	104	204	70	66	485	484	n.a.
Trade efficiency	27.40 %	11.22 %	12.00 %	4.88 %	0.15 %	−7.46 %	−3.63 %	−25.52 %	−3.84 %	−24.28 %	−5.62 %	−14.51 %	n.a.
Avg. profit/avg. loss	0.45	0.56	0.85	0.67	1.36	1.29	2.03	3.70	2.15	2.24	1.71	2.08	n.a.
Profitable trades	75 %	59 %	60 %	58 %	50 %	46 %	44 %	20 %	47 %	26 %	44 %	34 %	n.a.

The data cover from January 2000 to December 2013. A “performance” is a percentage measure of how much profit or loss the trading rule generated based on initial equity. An “annualized performance” calculates a performance over a year. It equals to a performance multiplied by 365 and divided by the number of days in the simulation. “Highest Open Drawdown” (HOD) is the maximum distance the equity line fell below the initial investment. A “buy and hold index” shows the percentage of the trading system’s profits when compared to a buy-and-hold strategy’s profits. A “profit and loss index” compares the amount of “Net Profit” (Trade Profit − Trade Loss) to the amount of winning or losing trades. Profit and Loss Index = 100 × (Net Profit)/[Max(Trade Profit, Trade Loss)]. A “reward and risk index” compares risk to reward. In this case, risk is defined as the “Highest Open Drawdown” (HOD) plus positive net profit, whereas reward is defined as the “Net Profit” (Trade Profit − Trade Loss) from the trading system. Reward and Risk Index = 100x(Net Profit)/[Max(Net Profit,0) + HOD]. A “trade efficiency” for long only strategy is calculated as (Exit price − Entry price)/(Highest price − Lowest price). A “trade efficiency” for short only strategy is calculated as (Entry price − Exit price)/(Highest price − Lowest price). An “average profit/average loss” is a ratio of average profit of profitable trades over average loss of unprofitable trades. MACD’s parameter 1, 2 and 3 are optimized over 10–15, 20–30 and 5–15, respectively. RSI’s parameter 1 is optimized over 5–25. STO’s parameter 1 and 2 are optimized over 5-15 and 1-5, respectively. STOD’s parameter 1, 2 and 3 are optimized over 5–15, 1–5 and 3–5, respectively

**Table 15:** Results from JSX Composite index (Indonesia) with standard parameters compared to optimized parameters

	RSI		STOCH		STOCH-D		MACD		DMI		OBV		Buy and hold (BH)
	Long	Short	Long	Short	Long	Short	Long	Short	Long	Short	Long	Short	
Standard parameters													
N1	14	14	5	5	5	5	12	12	14	14	3	3	n.a.
N2	n.a.	n.a.	1	1	1	1	26	26	n.a.	n.a.	n.a.	n.a.	n.a.
N3	n.a.	n.a.	n.a.	n.a.	3	3	9	9	n.a.	n.a.	n.a.	n.a.	n.a.
Performance	−7.45 %	−94.33 %	82.14 %	−83.80 %	1,391.21 %	62.70 %	396.61 %	−6.85 %	378.26 %	23.21 %	863.60 %	15.96 %	517.05 %
Annualized performance	−0.53 %	−6.74 %	5.87 %	−5.99 %	99.39 %	4.48 %	28.33 %	−0.49 %	27.02 %	1.66 %	61.70 %	1.14 %	36.94 %
Highest open drawdown (HOD)	50.35 %	95.19 %	46.08 %	87.45 %	16.79 %	1.64 %	18.48 %	20.36 %	2.05 %	8.50 %	19.89 %	0.00 %	49.48 %
Buy and hold index	−101.44 %	−118.24 %	−84.11 %	−116.21 %	169.07 %	−87.87 %	−23.29 %	−101.32 %	−26.84 %	−95.51 %	67.02 %	−96.91 %	0.00 %
Profit/loss index	−7.49 %	−78.32 %	27.41 %	−40.08 %	35.80 %	6.26 %	44.32 %	−1.32 %	72.19 %	17.75 %	38.88 %	4.14 %	100.00 %
Reward/risk index	−14.79 %	−99.10 %	64.06 %	−95.82 %	98.81 %	97.45 %	95.55 %	−33.65 %	99.46 %	−73.19 %	97.75 %	100.00 %	91.27 %
Total trades	15	21	174	175	690	690	130	241	60	57	331	331	n.a.
Trade efficiency	23.26 %	−20.51 %	14.88 %	2.39 %	6.81 %	−11.59 %	−1.61 %	−22.60 %	6.18 %	−17.58 %	−1.53 %	−17.92 %	n.a.
Avg. profit/avg. loss	0.34	0.43	0.78	0.44	1.54	1.82	2.37	3.19	3.60	1.93	2.00	2.38	n.a.
Profitable trades	73 %	33 %	64 %	58 %	50 %	37 %	43 %	24 %	50 %	39 %	45 %	31 %	n.a.
Optimal parameters													
N1	23	11	5	5	14	14	10	11	5	5	2	2	n.a.
N2	n.a.	n.a.	1	1	1	1	29	26	n.a.	n.a.	n.a.	n.a.	n.a.
N3	n.a.	n.a.	n.a.	n.a.	4	4	5	9	n.a.	n.a.	n.a.	n.a.	n.a.
Performance	39.22 %	−92.52 %	82.14 %	-83.80 %	2,278.45 %	125.70 %	528.91 %	7.52 %	443.81 %	35.77 %	1688.09 %	97.13 %	517.05 %
Annualized performance	2.80 %	−6.61 %	5.87 %	−5.99 %	162.78 %	8.98 %	37.79 %	0.54 %	31.71 %	2.56 %	120.60 %	6.94 %	36.94 %
Highest open drawdown (HOD)	40.66 %	93.98 %	46.08 %	87.45 %	8.13 %	0.00 %	13.03 %	9.15 %	7.10 %	8.50 %	18.44 %	1.86 %	49.48 %
Buy and hold index	−92.41 %	−117.89 %	−84.11 %	−116.21 %	340.66 %	−75.69 %	2.29 %	−98.55 %	−14.16 %	−93.08 %	226.48 %	−81.21 %	0.00 %
Profit/loss index	47.11 %	−71.90 %	27.41 %	−40.08 %	45.39 %	12.03 %	44.73 %	1.19 %	61.07 %	15.98 %	36.77 %	9.44 %	100.00 %
Reward/risk index	49.10 %	−98.44 %	64.06 %	−95.82 %	99.64 %	100.00 %	97.60 %	45.10 %	98.43 %	80.79 %	98.92 %	98.12 %	91.27 %
Total trades	7	28	174	175	551	551	167	244	135	135	777	777	n.a.
Trade efficiency	31.63 %	−10.77 %	14.88 %	2.39 %	6.99 %	−13.57 %	−2.06 %	−22.78 %	5.41 %	−17.29 %	2.93 %	−12.77 %	n.a.
Avg. profit/avg. loss	0.76	0.37	0.78	0.44	1.72	2.01	2.22	3.32	2.32	2.46	1.84	2.03	n.a.
Profitable trades	71 %	43 %	64 %	58 %	52 %	36 %	45 %	23 %	53 %	33 %	46 %	35 %	n.a.

The data cover from January 2000 to December 2013. A “performance” is a percentage measure of how much profit or loss the trading rule generated based on initial equity. An “annualized performance” calculates a performance over a year. It equals to a performance multiplied by 365 and divided by the number of days in the simulation. “Highest Open Drawdown” (HOD) is the maximum distance the equity line fell below the initial investment. A “buy and hold index” shows the percentage of the trading system’s profits when compared to a buy-and-hold strategy’s profits. A “profit and loss index” compares the amount of “Net Profit” (Trade Profit − Trade Loss) to the amount of winning or losing trades. Profit and Loss Index = 100 × (Net Profit)/[Max(Trade Profit, Trade Loss)]. A “reward and risk index” compares risk to reward. In this case, risk is defined as the “Highest Open Drawdown” (HOD) plus positive net profit, whereas reward is defined as the “Net Profit” (Trade Profit − Trade Loss) from the trading system. Reward and Risk Index = 100 × (Net Profit)/[Max(Net Profit,0) + HOD]. A “trade efficiency” for long only strategy is calculated as (Exit price − Entry price)/(Highest price − Lowest price). A “trade efficiency” for short only strategy is calculated as (Entry price − Exit price)/(Highest price − Lowest price). An “average profit/average loss” is a ratio of average profit of profitable trades over average loss of unprofitable trades. MACD’s parameter 1, 2 and 3 are optimized over 10–15, 20–30 and 5–15, respectively. RSI’s parameter 1 is optimized over 5-25. STO’s parameter 1 and 2 are optimized over 5–15 and 1–5, respectively. STOD’s parameter 1, 2 and 3 are optimized over 5–15, 1–5 and 3–5, respectively

**Table 16:** Results from PSE composite index (the Philippines) with standard parameters compared to optimized parameters

	RSI		STOCH		STOCH-D		MACD		DMI		OBV		Buy and hold (BH)
	Long	Short	Long	Short	Long	Short	Long	Short	Long	Short	Long	Short	
Standard parameters													
N1	14	14	5	5	5	5	12	12	14	14	3	3	n.a.
N2	n.a.	n.a.	1	1	1	1	26	26	n.a.	n.a.	n.a.	n.a.	n.a.
N3	n.a.	n.a.	n.a.	n.a.	3	3	9	9	n.a.	n.a.	n.a.	n.a.	n.a.
Performance	−40.04 %	−91.59 %	147.58 %	−37.66 %	1176.80 %	213.29 %	397.85 %	457.66 %	87.21 %	−17.88 %	27.02 %	−1.74 %	175.49 %
Annualized performance	−2.86 %	−6.55 %	10.55 %	−2.69 %	84.11 %	15.24 %	28.43 %	32.71 %	6.23 %	−1.28 %	13.60 %	−0.88 %	12.54 %
Highest open drawdown (HOD)	65.39 %	92.95 %	54.97 %	50.51 %	28.75 %	3.84 %	3.20 %	1.45 %	23.61 %	25.83 %	2.64 %	17.80 %	52.70 %
Buy and hold index	−122.82 %	−152.19 %	−15.90 %	−121.46 %	570.58 %	21.54 %	126.71 %	160.79 %	−50.30 %	−110.19 %	−13.17 %	−105.59 %	0.00 %
Profit/loss index	−51.85 %	−94.43 %	37.61 %	−15.00 %	45.83 %	19.44 %	55.91 %	25.81 %	49.28 %	−14.96 %	39.52 %	−5.31 %	100.00 %
Reward/risk index	−61.23 %	−98.53 %	72.86 %	−74.56 %	97.62 %	98.23 %	99.20 %	99.68 %	78.64 %	−69.21 %	91.11 %	−9.77 %	76.91 %
Total trades	13	16	205	206	676	677	118	183	71	70	56	55	n.a.
Trade efficiency	2.93 %	−31.56 %	17.17 %	4.68 %	2.35 %	−5.49 %	0.39 %	−14.95 %	−11.26 %	−18.97 %	1.26 %	−18.37 %	n.a.
Avg. profit/avg. loss	0.41	0.12	0.83	0.66	1.98	1.72	2.51	3.79	2.86	1.53	2.05	2.12	n.a.
Profitable trades	54 %	31 %	66 %	56 %	48 %	42 %	47 %	26 %	41 %	36 %	45 %	31 %	n.a.
Optimal parameters													
N1	6	5	6	6	15	15	13	14	10	10	3	3	n.a.
N2	n.a.	n.a.	1	1	1	1	28	24	n.a.	n.a.	n.a.	n.a.	n.a.
N3	n.a.	n.a.	n.a.	n.a.	4	4	9	8	n.a.	n.a.	n.a.	n.a.	n.a.
Performance	16.37 %	−75.81 %	168.13 %	−34.01 %	2,070.96 %	431.77 %	491.93 %	533.57 %	156.67 %	14.62 %	68.92 %	24.88 %	175.49 %
Annualized performance	1.17 %	−5.42 %	12.02 %	−2.43 %	148.01 %	30.86 %	35.16 %	38.13 %	11.20 %	1.04 %	34.70 %	12.53 %	12.54 %
Highest open drawdown (HOD)	55.33 %	79.05 %	46.97 %	46.90 %	18.01 %	0.00 %	2.91 %	0.00 %	20.30 %	20.53 %	0.00 %	11.00 %	52.70 %
Buy and hold index	−90.67 %	−143.20 %	−4.19 %	−119.38 %	1,080.10 %	146.04 %	180.32 %	204.05 %	−10.72 %	−91.67 %	121.47 %	−20.05 %	0.00 %
Profit/loss index	10.88 %	−40.60 %	38.84 %	−13.15 %	54.75 %	28.88 %	58.54 %	26.79 %	62.70 %	10.15 %	60.92 %	38.14 %	100.00 %
Reward/risk index	22.83 %	−95.90 %	78.16 %	−72.50 %	99.14 %	100.00 %	99.41 %	100.00 %	88.53 %	41.60 %	100.00 %	69.34 %	76.91 %
Total trades	59	81	180	180	526	525	118	184	78	77	89	88	n.a.
Trade efficiency	23.84 %	8.01 %	19.63 %	5.81 %	4.01 %	−5.36 %	0.42 %	−14.62 %	−6.24 %	−14.24 %	11.20 %	−6.92 %	n.a.
Avg. profit/avg. loss	0.42	0.45	0.68	0.65	2.33	1.86	2.67	3.76	3.29	1.84	1.91	2.83	n.a.
Profitable trades	73 %	57 %	71 %	57 %	49 %	43 %	47 %	27 %	45 %	38 %	57 %	36 %	n.a.

The data cover from January 2000 to December 2013. Due to a data limitation, results of the OBV trading strategy is based on the sample from January 2012 to December 2013. A performance of the BH strategy over the same period is 31.12 %. A “performance” is a percentage measure of how much profit or loss the trading rule generated based on initial equity. An “annualized performance” calculates a performance over a year. It equals to a performance multiplied by 365 and divided by the number of days in the simulation. “Highest Open Drawdown” (HOD) is the maximum distance the equity line fell below the initial investment. A “buy and hold index” shows the percentage of the trading system’s profits when compared to a buy-and-hold strategy’s profits. A “profit and loss index” compares the amount of “Net Profit” (Trade Profit − Trade Loss) to the amount of winning or losing trades. Profit and Loss Index = 100 × (Net Profit)/[Max(Trade Profit, Trade Loss)]. A “reward and risk index” compares risk to reward. In this case, risk is defined as the “Highest Open Drawdown” (HOD) plus positive net profit, whereas reward is defined as the “Net Profit” (Trade Profit − Trade Loss) from the trading system. Reward and Risk Index = 100x(Net Profit)/[Max(Net Profit,0) + HOD]. A “trade efficiency” for long only strategy is calculated as (Exit price − Entry price)/(Highest price − Lowest price). A “trade efficiency” for short only strategy is calculated as (Entry price − Exit price)/(Highest price − Lowest price). An “average profit/average loss” is a ratio of average profit of profitable trades over average loss of unprofitable trades. MACD’s parameter 1, 2 and 3 are optimized over 10–15, 20–30 and 5–15, respectively. RSI’s parameter 1 is optimized over 5–25. STO’s parameter 1 and 2 are optimized over 5–15 and 1–5, respectively. STOD’s parameter 1, 2 and 3 are optimized over 5–15, 1–5 and 3–5, respectively

Noticeably, unprofitable strategies like RSI and STOCH still perform worse than a simple Buy-and-Hold strategy (BH) even with optimized parameters except in the Singaporean market, where they manage to beat a BH with the long-only strategies. The riskiness as measured by the Highest Open Drawdown (HOD) tends to decrease, but in certain cases, it marginally increases. The DMI long-only trading strategy (with optimized parameters) is profitable than a BH strategy in the Thai, Malaysian and Singaporean markets. Profits increase further with optimized parameters. However, it is still not as profitable as a BH strategy in the Indonesian and the Philippine markets.

Profitable strategies like STOCH-D, MACD and OBV perform much better with optimized parameters. For example, the MACD long-only trading strategy with optimized parameters has an annualized performance of 45.06 % compared to 28.65 % with standard parameters for the Thai market. In addition, the riskiness as measured by HOD decreases significantly from 10.44 to 4.11 %. Like in the above case, the HOD tends to decrease in general but it may in fact increase slightly in certain cases.

In short, unprofitable strategies (when compared to a BH) like RSI and STOCH generally give lower returns than that from a BH even with optimized parameters. Profitable strategies (when compared to a BH) like MACD, STOCH-D and OBV with optimized parameters yield even better average returns with generally lower risk as measured by HOD.

Very interestingly, optimized parameters do not improve the odds of profitable trades. The percentages of profitable trades remain relatively the same. It may even decrease in some cases. Again, this result seems to validate the idea that profitable strategies make money not from correctly predicting directions of the market, but from letting the profits to run while minimizing losses.

The optimized parameter values differ from standard parameter values. For profitable strategies like STOCH-D, MACD and OBV, optimized values would drastically increase investment returns. This result strongly suggests that traders should optimize parameters of their trading strategies through back testing rather than stick with the textbook standard parameters. The back testing must also be done specifically for each market. The optimized parameters from one market may not work that well in another market.

## Conclusions

This paper studies the profitability of technical trading strategies when applied to five Southeast Asian stock market indices: SET index (Thailand), FTSE Bursa Malaysia KLC index (Malaysia), FTSE Straits Times index (Singapore), JSX Composite index (Indonesia), and PSE composite index (the Philippines). The data cover a period of 14 years from January 2000 to December 2013. The results are then compared to a simple Buy-and-Hold (BH) strategy.

Overall, our empirical results show that these five Southeast Asian stock markets are, to a varying degree, at least close to weak-form efficient as most popular technical trading strategies could not earn statistically significant returns, particularly after transaction costs. The only exception is the Thai market. Nevertheless, certain technical strategies like MACD, STOCH-D or more sophisticated strategies may still provide net excess returns. Our results also suggest that traders should optimize parameters of their trading strategies rather than stick with standard textbook parameters.

Though the topic of profitability of technical analysis has been widely investigated as summarized in Park and Irwin (2007), most studies focus on statistical tests of returns from technical trading and overlook other performance measures. By using both formal statistical tests and technical trading performance measures, this paper finds three new insights not mentioned in previous studies.

Firstly, in terms of market timing, we find that using technical indicators does not help much. The trade efficiency measures, our indicators of market timing ability, are normally low with few exceptions. The implication is that even with seemingly profitable technical trading strategies, traders cannot expect to buy at a relatively low price and sell at a relatively high price by just using technical trading rules.

Secondly, technical trading rules and indicators help not so much in terms of market timing but in terms of countering behavior biases. Individual investors have the behavioral bias called disposition effect as they tend to sell winning stocks too soon and holding on to losing stocks too long (Odean 1998). Technical trading rules help to counter this bias by allowing profits to run in profitable trades while cutting losses in unprofitable ones. That is how profitable strategies like MACD and STOCH-D beat a Buy-and-Hold (BH) strategy. The implication is that even if the market is weak-form efficient, the use of technical trading rules may still be beneficial to individual investors as it counters the above bias.

Thirdly, even profitable strategies such as MACD and STOCH-D could not reliably predict subsequent market directions as their profitable trades are usually less than fifty percent of total number of trades. They make money from having a higher average profit from profitable trades than an average loss from unprofitable ones. Interestingly, optimized parameters do not improve the odds of profitable trades. Our results support the idea that profitable strategies make money not from outguessing market directions, but from maximizing average profits and minimizing average losses.

The limitation of our study is that we cover only the more popular technical trading strategies with standard parameters. Future studies could extend to include more technical indicators with different parameters. In addition, there was a generally strong uptrend in our sample, future studies may attempt to include other market situations in the samples. In terms of testing instruments, this paper tests technical trading systems only on the market indices. Future studies can extend the coverage to individual sectors or stocks as the performances of technical trading rules may vary across sectors or stock characteristics.
